# Exploring the Lesser-Known Bioactive Natural Products of Plant Species of the Genus *Cannabis* L.: Alkaloids, Phenolic Compounds, and Their Therapeutic Potential

**DOI:** 10.3390/plants14091372

**Published:** 2025-04-30

**Authors:** Raphaël Boucher, Hugo Germain, Isabel Desgagné-Penix

**Affiliations:** Department of Chemistry, Biochemistry and Physics, Université du Québec à Trois-Rivières, Trois-Rivières, QC G9A 5H7, Canada; raphael.boucher@uqtr.ca (R.B.); hugo.germain@uqtr.ca (H.G.)

**Keywords:** nitrogen-containing compounds, spermidine-type alkaloids, non-cannabinoid metabolites, plant specialized metabolism, cannabisativin, flavonoids, stilbenes, cannflavins, cannabisins

## Abstract

Plant species of the genus *Cannabis* L. are predominantly recognized for their cannabinoids, which have garnered significant attention due to their bioactive properties. However, *Cannabis* also produces a diverse array of bioactive compounds with promising pharmacological potential that remain underexplored. This review focuses primarily on phytochemicals derived from *Cannabis sativa* L. subspecies, including both its drug-type and fiber-type varieties, which are the most widely cultivated and studied within the genus. Among these, nitrogen-containing compounds such as spermidine alkaloids exhibit neuroprotective and anti-aging properties, while hydroxycinnamic acids and hydroxycinnamic acid amides, including N-trans-caffeoyltyramine and N-trans-feruloyltyramine, have demonstrated notable antioxidant and anti-inflammatory activities. Additionally, *Cannabis* species are a valuable source of unique stilbenes, such as canniprene, and flavonoids, including cannflavin A and B, which demonstrated potent anti-inflammatory and antiproliferative effects. Despite this rich phytochemical diversity, research on these compounds remains limited, largely due to historical legal restrictions. This literature review consolidates and updates current knowledge on these lesser-studied phytochemicals of *Cannabis*, detailing their biosynthetic pathways, metabolic precursors, and emerging therapeutic applications. By expanding the research focus beyond cannabinoids, this work aims to enhance our understanding of *Cannabis*’s full pharmacological potential and promote further investigation into its diverse chemical constituents.

## 1. Introduction

*Cannabis* L., a species historically cultivated for its fibers, oils, and psychoactive properties, has gained increasing interest due to its diverse pharmacological potential. The most notorious specialized metabolites from *Cannabis* are cannabinoids—meroterpenoids (i.e., partial terpenoid derivative, also known as terpenophenolics) [1,2,3,4]—which are predominantly found in female flowers, although trace amounts may be present in other tissues like leaves and stems, with seeds and roots generally lacking psychoactive cannabinoids [5,6]. Most *Cannabis* research has therefore focused on organs with high cannabinoid concentrations, primarily flowers and to some extent leaves, while seeds have been studied for their oil content and nutraceutical applications, and stems, for fiber. Consequently, this research focus has led to a targeted yet incomplete understanding of the biochemical processes governing *Cannabis* metabolism, with some plant organs remaining underexplored.

In light of longstanding controversies regarding the taxonomy of the *Cannabis* genus and its historical species designations (i.e., *sativa*, *indica*, and *ruderalis*), this review adopts the guidelines proposed by Pollio (2016) [7], which proposes to avoid the distinction between intraspecific taxa, and advises to apply a nomenclature system based on the International Code of Nomenclature for Cultivated Plants (ICNCP) [8].

While cannabinoids, such as tetrahydrocannabinol (THC) and cannabidiol (CBD), dominate the current understanding of *Cannabis* bioactivity, other specialized metabolites—including alkaloids, flavonoids, and stilbenes—also contribute to its pharmacological profile but remain less studied [2,9,10]. A CANNUSE database analysis report indicates that traditional usage of *Cannabis* is composed mainly of medicinal use (75% of reports), wherein leaves constitute 56% of medicinal reports [11]. Reported conditions for which *Cannabis* leaves were used include digestive system and nutritional disorders (157 reports), nervous system and mental disorders (131 reports), skin and subcutaneous tissue disorders (108 reports), infections and infestations (105 reports), and pain and inflammation (101 reports). Whilst cannabinoids may contribute to the pharmacological effects of leaves, flavonoids, stilbenes, and alkaloids are also reported in this tissue, and cannabinoids cannot possibly account for the treatment of all the listed conditions. The imputability of biological activity has not been determined, that is, we do not know the combined effect of these metabolites and how it translates to their respective pharmacological action. These molecules, which vary significantly in their structure, biosynthetic pathways, and bioactivities, may offer therapeutic potential as anti-inflammatory, antioxidant, neuroprotective, and antimicrobial agents [5,6]. The nitrogen-containing (NC) specialized metabolites in *Cannabis*, which include alkaloids cannabisativine and anhydrocannabisativine, exclusive as of yet to *Cannabis*, simple amines with valuable activity like hordenine, produced notably by barley, and amides commonly found in medicinal plants, namely coumaroyltyramine and derivatives, as well as cannabisins. These NC compounds are biosynthesized via pathways that differ from cannabinoid biosynthesis and often stem from the metabolism of amino acids or other nitrogenous precursors. Although NC metabolites are significantly less concentrated than cannabinoids, emerging research has identified notable bioactive properties worthy of therapeutic consideration such as neuroprotective, antitumor, and analgesic effects, suggesting they warrant further investigation [12,13,14].

In addition to NC compounds, *Cannabis* is also rich in non-nitrogenous phenolic compounds, notably flavonoids and stilbenes, which have demonstrated antioxidant, anti-inflammatory, and antimicrobial properties. Flavonoids like cannaflavin A and B, unique to *Cannabis*, demonstrated the notable inhibition of inflammatory pathways, with potency exceeding some NSAIDs [15,16,17]. Although stilbenes have been reported to be less abundant, they contribute to the plant’s antioxidative profile and may enhance the pharmacological effects of cannabinoids through synergistic interactions [18,19]. *Cannabis* stilbenes are numerous, and many are, to date, unique to *Cannabis*, namely canniprene, cannastilbenes (I, IIa, and IIb), and many spiroindans, including cannabispirone.

This narrative review aims to explore these lesser-known facets of *Cannabis*, with a particular focus on specialized nitrogen metabolism, with an emphasis on the metabolites in its roots—a largely neglected area in *Cannabis* research. The review will delve into the NC specialized metabolites and phenolic compounds, concentrating on phenolic acids and their derivatives, such as hydroxycinnamic acid amides and lignanamides, alongside flavonoids, stilbenes, spermidine alkaloids, and other nitrogenous molecules within *Cannabis*. In doing so, we will highlight recent advancements in the biosynthesis, biological activity, and potential therapeutic applications of these compounds. Furthermore, this review will address the current challenges and propose future research directions to better understand these lesser-known compounds, which could unlock novel therapeutic applications and enhance the medicinal versatility of *Cannabis* [20,21].

To produce this narrative review, PubMed database was searched for articles with terms such as cannabisativine (six articles considered), *Cannabis* flavonoids (nineteen), *Cannabis* roots (seven), *Cannabis* lignanamides (sixteen), *Cannabis* hydroxycinnamic acid amides (six), *Cannabis* flavonoid biosynthesis (thirteen), cannflavin biosynthesis (eleven), *Cannabis* stilbene biosynthesis (four), hordenine activity (thirteen), N-trans-coumaroyltyramine activity (nine), N-trans-caffeoyltyramine activity (seventeen), N-trans-feruloyltyramine activity (thirty-six), cannabisin activity (twenty-three), lignanamide activity (forty-four), cannflavin activity (nineteen), *Cannabis* stilbene activity (seven), denbinobin activity (eighteen), and canniprene activity (two). Well-known biosynthetic processes like aromatic amino acid biosynthesis, polyamine, and phenylpropanoid and flavonoid biosynthetic pathways were not subjected to the review process. Given the limited information on some sections of this review, no filter was used for the publication dates on all individual search criteria. However, when the number of published articles exceeded a reasonable amount (more than 50), only the most recent articles were selected (in the last 10 years). Articles were dismissed when activity assays were performed using plant extracts (both in vitro and in vivo), meaning only articles with purified compounds were considered. Figures were created using PerkinElmer’s ChemDraw (Prime level), version 22.2.0.3300. Molecular structures were designed according to their respective PubChem structures.

## 2. Generalities and Classification of Cannabis Metabolites

**Figure 1:**
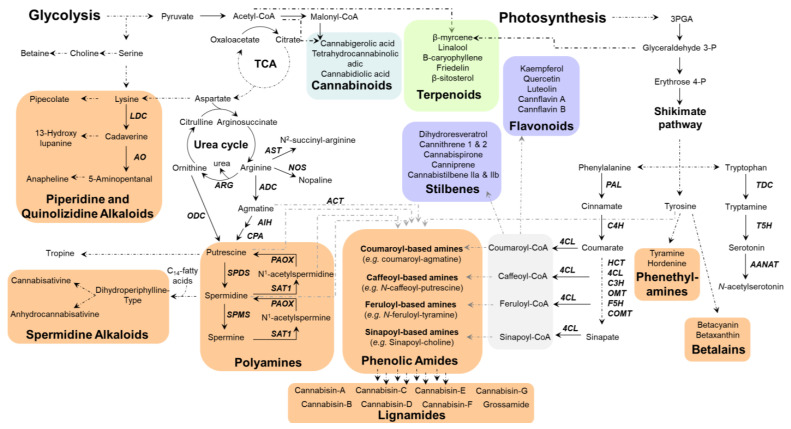
Overview of the primary to specialized metabolic flow in *Cannabis*. Plants synthesize metabolites through photosynthesis, which serves as foundational source for both primary and specialized metabolites. Primary metabolites, produced through essential pathways such as glycolysis, tricarboxylic acid cycle (TCA), shikimate pathway, and urea cycle, provide precursors for specialized metabolite biosynthesis. This diagram highlights key cannabinoids (blue), terpenoids (green), and nitrogen-containing (orange) and phenolic (purple) compound pathways, with examples of metabolites frequently found in *Cannabis* further detailed in Section 3. A solid arrow represents one enzymatic reaction (bold abbreviation next to it is the name of the enzyme), whereas a dashed arrow indicates sequences of multiple reactions. The figure is adapted from schemes by Flores-Sanchez and Verpoorte (2008) [29]. Abbreviations are found in Appendix A—Table A1, Table A2, Table A3, Table A4 and Table A5.

The general metabolism of living organisms encompasses the biochemical reactions and resulting molecules essential for survival, growth, and reproduction [22]. This metabolism is composed of numerous interconnected pathways, including glycolysis, the tricarboxylic acid cycle (TCA), the urea cycle, and the shikimate pathway, which supports the anabolism of aromatic amino acids (Figure 1) [22]. Certain general metabolic pathways remain remarkably conserved across diverse life forms, underscoring their fundamental role and efficiency [22,23]. Polyamines are an example of ubiquitous metabolites possessing a widely conserved biosynthetic pathway.

A polyamine is an aliphatic molecule containing more than one nitrogen atom in its structure. They are classified according to their number of nitrogen atoms: diamines (2 N, e.g., putrescine), triamines (3 N, e.g., spermidine), and tetraamines (4 N, e.g., spermine) [24]. Due to their structure, polyamines have a strong nucleophilic potential and a hydrophilic character, possessing a primary amine on each end of their carbon chain. Their primary amines are also ionizable at physiological pH, conferring a cationic potential comparable to that of a calcium atom (Ca^2+^) or a magnesium atom (Mg^2+^) [24]. However, unlike those atoms, the charge is distributed along a carbon skeleton, rather than in a single divalent atom, allowing hydrogen and electrostatic and hydrophobic interactions to stabilize or destabilize, depending on the nature of the charges and the macromolecules in the immediate vicinity [24]. Such a mechanism is fundamental in the ability to adapt to environmental stimuli, affecting crucial processes, namely RNA/DNA polymerase activity, DNA integrity from depurination by heat, as well as acting as an ROS-scavenging entity [25]. In addition to essential metabolites, organisms biosynthesize specialized metabolites (also known as secondary metabolites, implying a hierarchical importance of the molecules found in a given cell, while simultaneously disregarding the environment-specific nature of these metabolites), which offer ecological advantages that support survival and the transmission of genes to future generations. Specialized metabolites, which reflect an organism’s adaptation to its environment, serve various ecological functions, ranging from attracting pollinators, to protection against pathogens and UV rays, to repulsion of herbivores and other predators [26]. Obviously, these metabolites facilitate the survival of the organisms that produce them, but they also are useful for the survival of other organisms, including humans, by providing antimicrobial, antioxidant, anti-inflammatory, and anti-cancer activities [27,28].

In plants, specialized metabolites are generally classified into three main groups based on their chemical structure and biosynthetic origin: alkaloids, terpenoids, and phenolic compounds (Figure 1) [28]. Among these, NC compounds, a diverse category of plant metabolites with nitrogen atoms in their molecular structure, include alkaloids, simple amines, amides, and certain peptides. NC metabolites play crucial roles in plant defense, growth regulation, and ecological interactions with herbivores, pathogens, and symbiotic organisms. Furthermore, these bioactive compounds have significant pharmacological potential, making them highly valuable for therapeutic applications.

### 2.1. Phenolic Compounds of Cannabis

Phenolic compounds encompass a broad group of specialized metabolites, characterized by a hydroxyl group attached to a benzene ring (phenol). This structural foundation allows for substantial chemical diversity, encompassing, among others, simple phenols, phenolic acids, cinnamic acids, flavonoids, and stilbenes (Table 1). Additionally, this group includes complex phenolic polymers like lignans, tannins, and phlobaphenes, which contribute to structural integrity [28]. Various subclasses of phenolic compounds have been identified in *Cannabis*, such as is the case with flavonoids, underscoring the structural and functional diversity within this group [30].

The roles of phenolic compounds in plants are as diverse as their structures. Their antioxidative properties, for instance, are integral to plant defense, where they capture reactive oxygen species (ROS) to manage cellular ROS such as hydrogen peroxide (H_2_O_2_) concentrations [31]. Phenolic compounds also protect plants from UV radiation, as exemplified by flavonoids and cannabinoids in *Cannabis*, and they defend against aggression by predators, herbivores, and pathogens. Phenolic compounds are also responsible for several colors found in the plant kingdom; this is particularly the case for the vibrant hues of anthocyanins, a subgroup of flavonoids [12,31,32,33]. Furthermore, phenolic polymers biosynthesized from phenylpropanoids (e.g., lignin, suberin, condensed tannins) provide rigidity and stability to gymnosperms and angiosperms, particularly against mechanical stresses [34], whereas other metabolites also derived from the phenylpropanoid pathway contribute to pathogen resistance, promoting the colonization of new environments [35]. Although phenolic compounds’ function is understood in a general sense, all in all, not many studies investigate the roles of *Cannabis* phenolics in planta.

### 2.2. Alkaloids of Cannabis

The classification of alkaloids is complex, and several approaches exist, each with its own rationale. Some classify alkaloids according to their chemical structure, others based on the taxonomic group of the producing organism, or as is often preferred, according to their biosynthetic origin pathway. Alkaloids are molecules containing at least one nitrogen atom and generally have a basic nature, hence their name—alkali—from Arabic, *al-qali* [36]. Generally, alkaloids are derived from amino acids, but some alkaloids are biosynthesized via transamination reactions [36].

According to the chemical structures’ classification, heterocyclic alkaloids can be distinguished from aliphatic ones [37]. Aliphatic alkaloids have their nitrogen atom in an open carbon chain, while heterocyclic alkaloids have theirs in a closed chain (a cycle). Plants can generate several alkaloids, which is what the taxonomic classification of alkaloids is based on. According to this classification, alkaloids produced by a common taxon can be identified as such, for example *Cannabinaceous* alkaloids, referring to any alkaloid produced by members of the *Cannabaceae* plant family, including *Cannabis* [37]. However, this approach, nonetheless useful for phylogeny, is less appropriate when considering alkaloids as a chemical species, since alkaloids produced by the same organism can be wildly different from one another.

Classification according to the biosynthetic pathways provides more precision when compared to the two approaches described above. It could allow for the distinguishment of alkaloids according to their nature (nitrogen of amino acid or transamination) and their chemical type (type of carbon chain). This then allows for the differentiation of purine alkaloids from true alkaloids. A true alkaloid is defined as an alkaloid whose biosynthesis involves the catabolism of amino acids, where the nitrogen atom of the latter is included in the heterocycle of the alkaloid. Purines are not derived from amino acids; despite the nitrogen heterocycles, they would not be considered true alkaloids. Thus, alkaloids obtained from transamination would be referred to as pseudo-alkaloids. There also exists what would be referred to as proto-alkaloids, derived from amino acids, but with an aliphatic nitrogen, which are fairly common in *Cannabis* (Table 2) [36].

In the *Cannabis* plant, we find, among others, piperidine, pyridine, pyrrolidine, and spermidine alkaloids, phenylethylamines, and tertiary/quaternary amines. In plants, alkaloids and other specialized NC metabolites play critical roles in defense against pathogens, herbivores, and other predators, often repelling or killing these threats. In some cases, alkaloids also act as regulators of growth and development [38]. The role of *Cannabis* alkaloids for the plant remains a mystery.

### 2.3. Specialized Metabolites of Cannabis

**Figure 2:**
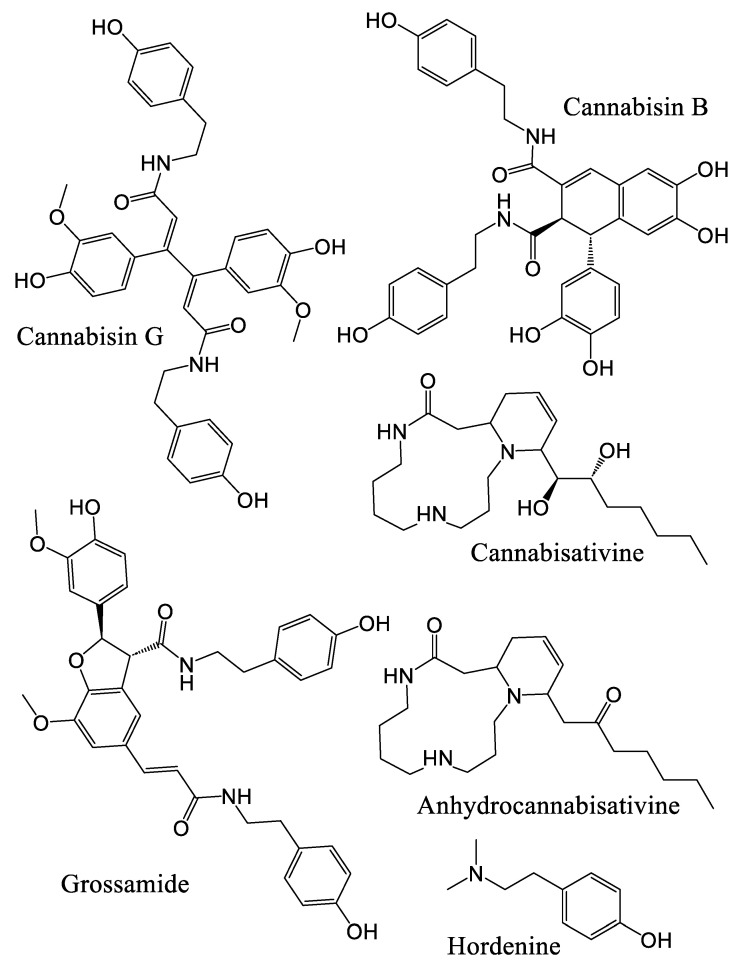
Examples of nitrogen-containing specialized metabolites identified in *Cannabis*.

To date, several NC specialized metabolites have been identified in *Cannabis*, located in various parts of the plants, including roots, leaves, stems, seeds, and pollen [29,39]. Identified quaternary amines include choline, trigonelline, _L_-isoleucine betaine, neurine, and muscarine, although the presence of muscarine in the roots remains uncertain [29]. These amines are derived from amino acids, similarly to piperidine, pyrrolidine, and hordenine, a phenylethylamine that is also found in *Cannabis* (Figure 1 and Figure 2).

The spermidine-type alkaloids reported in *Cannabis* include cannabisativine and anhydrocannabisativine (Figure 1 and Figure 2) [13,21,30,40]. In total, ten alkaloids have been identified in *Cannabis*, primarily found in the roots, but also detected in other parts of the plant [29]. Additionally, fifteen phenolic amides and lignanamides have been identified in *Cannabis* (Figure 1 and Figure 2). Among these are the hydroxycinnamic acid amides (HCAAs) N-trans-coumaroyl-tyramine in roots, and N-trans-feruloyltyramine and N-trans-caffeoyltyramine in flowers, seeds, and roots. Other metabolites include cannabisins A to G, as well as cannabisins M, N and O, 3,3′-demethylheliotropamide, and grossamide, with cannabisins B and G present in the roots [12,14,29,30,41]. Cannabisins, 3,3′-demethylheliotropamide and grossamide are HCAA dimers, as shown in Figure 2.

While flavonoids and stilbenes lack a nitrogen atom in their structures, they are derived from the phenylpropanoid pathway and exhibit biological activities of medical interest. These metabolites are primarily found in the leaves and flowers of *Cannabis*. Notable flavonoids include cannflavin A, B, and C, whilst at least thirty distinct stilbenes have been identified [30,42].

*Cannabis* uses a variety of metabolic pathways to produce these specialized metabolites (Figure 1 and Figure 2). In the following sections, we will explore these pathways, examining the biosynthetic routes that enable the production of both alkaloids and phenolic compounds.

## 3. Biosynthetic Routes to Nitrogen-Containing and Phenolic Metabolites in Cannabis

The gene sequences corresponding to the enzymes discussed in the following sections are listed in Appendix A—Table A1, Table A2, Table A3, Table A4 and Table A5.

### 3.1. Plant Aromatic Amino Acid Biosynthesis

**Figure 3:**
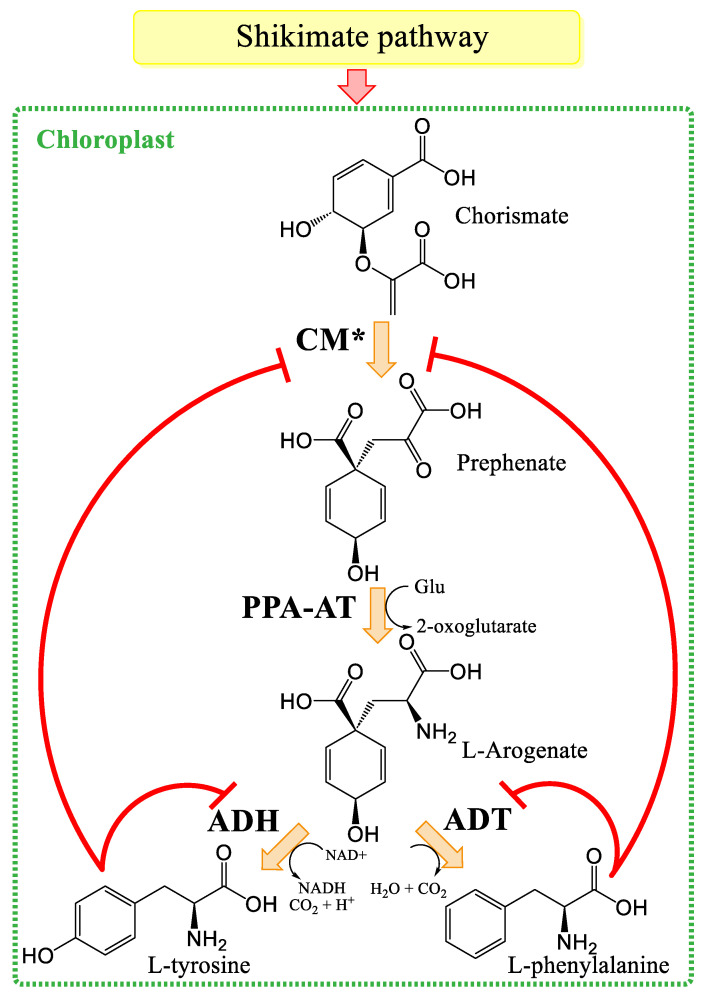
Aromatic amino acid biosynthetic pathway in plants. CM, chorismate mutase (* engaging step of the pathway). Arrow represents one biochemical reaction. PPA-AT, prephenate aminotransferase. ADT, arogenate dehydratase. ADH, arogenate dehydrogenase. In red: inhibition regulatory pathways. Glu, glutamate. Chorismate is converted to prephenic acid by the enzyme chorismate mutase (E.C. 5.4.99.5). Prephenate undergoes transamination by the enzyme prephenate aminotransferase (E.C. 2.6.1.79), using glutamate, producing L-arogenate and 2-oxoglutarate. The enzyme arogenate dehydratase (E.C. 4.2.1.91) completes the aromatic ring of the L-arogenate cycle by removing the hydroxyl and carboxylic groups attached to it, thus forming phenylalanine and generating H_2_O and CO_2_. To obtain tyrosine, arogenate is oxidized by the enzyme arogenate dehydrogenase (E.C. 1.3.1.43) with the cofactor NAD+, producing a molecule of CO_2_ and one of NADH.

Aromatic amino acids are precursors of a range of phenolic compounds. They act as intermediates between general and specialized metabolism. It is therefore appropriate to study their biosynthesis, which occurs via the shikimate pathway. This pathway uses intermediates from the pentose phosphate and glycolysis pathways to produce chorismic acid in plastids, which is the precursor of all aromatic amino acids (Figure 3) [43,44,45].

### 3.2. General Phenylpropanoids and Phenolic Acids Biosynthesis

**Figure 4:**
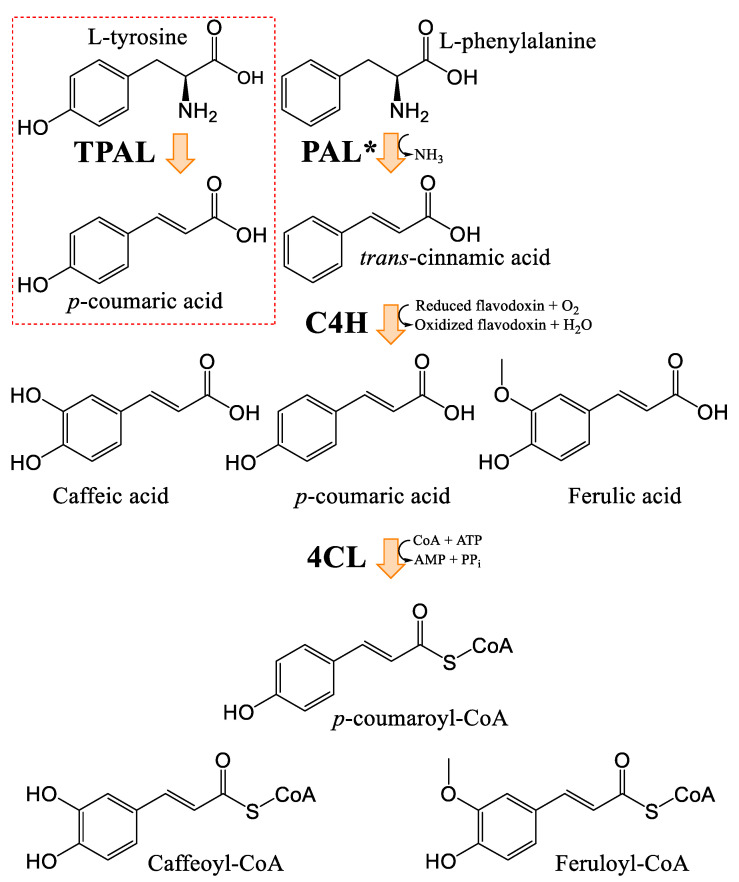
General phenylpropanoid pathway up to cinnamic acid thioesters’ biosynthesis. (* engaging step of the pathway). Red-dotted box: alternative pathway for *p*-coumaric acid biosynthesis. Arrow represents one biochemical reaction. PAL, phenylalanine ammonia-lyase. TPAL, tyrosine/phenylalanine ammonia-lyase. C4H, cinnamate 4-hydroxylase. 4CL, 4-coumarate-CoA-ligase. Phenylalanine will undergo deamination by the enzyme phenylalanine ammonia-lyase (E.C. 4.3.1.24), which generates a molecule of NH_3_ and trans-cinnamic acid. Tyrosine/phenylalanine ammonia-lyase (E.C. 4.3.1.25) can realise the same reaction as PAL and can also convert tyrosine into *p*-coumaric acid. Cinnamate 4-hydroxylase (E.C. 1.14.14.91) adds a hydroxyl group to the *para* position of trans-cinnamic acid, generating *p*-coumaric acid. 4-coumarate-CoA-ligase (E.C. 6.2.1.12) uses an ATP molecule to convert *p*-coumaric acid to *p*-coumaroyl-CoA, releasing an AMP and a pyrophosphate molecule.

The biosynthesis of phenolic compounds occurs via the phenylpropanoid pathway, starting with phenylalanine (Figure 4). This first step regulates the flow of metabolites entering this pathway [46]. Tyrosine/phenylalanine ammonia-lyase, in some dicotyledonous organisms, and particularly monocotyledons, can catalyze the same reaction as PAL, but can also use tyrosine as a substrate [47,48]. Otherwise, *p*-coumaric acid is generated by the activity of the cinnamate 4-hydroxylase. Other cinnamic acid derivatives have been identified in plants, including caffeic acid, sinapic acid, and ferulic acid [45,46]. *p*-coumaroyl-CoA will serve as precursor for most phenolic compounds [46] and *p*-coumaric acid analogues can also serve as ligands for 4CL.

### 3.3. Hydroxycinnamic Acids and Lignanamides Biosynthesis

**Figure 5:**
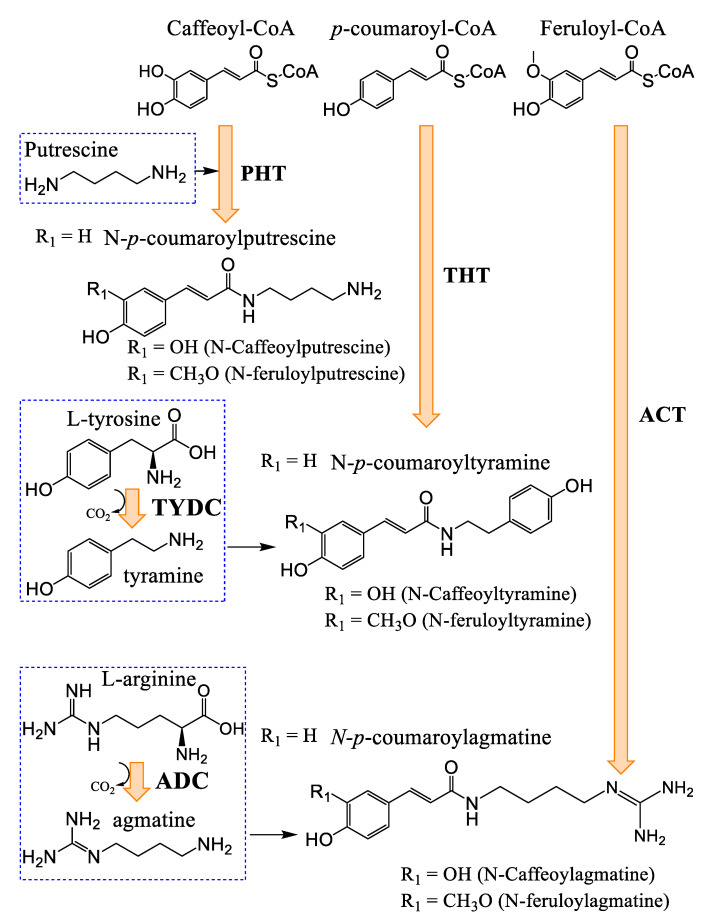
General biosynthesis of hydroxycinnamic acid amides from cinnamic acid thioesters. Blue-dotted boxes: some aminated molecules that can be condensed on a cinnamic acid moiety. Arrow represents one biochemical reaction. TYDC, tyrosine decarboxylase. ADC, arginine decarboxylase. PHT, putrescine N-hydroxycinnamoyltransferase. THT, hydroxycinnamoyl-CoA: tyramine N-(hydroxycinnamoyl)transferase. ACT, agmatine N4-coumaroyltransferase. Tyrosine decarboxylase (E.C. 4.1.1.25) produces tyramine and CO_2_ from tyrosine. Tryptamine decarboxylase (E.C. 4.1.1.105) produces tryptamine and CO_2_ from tryptophan. Putrescine *N*-hydroxycinnamoyltransferase (E.C. 2.3.1.138) produces a putrescine-derived hydroxycinnamic amide (e.g., *N*-*p*-coumaroylputrescine). Hydroxycinnamoyl-CoA:tyramine *N*-(hydroxycinnamoyl) transferase (E.C. 2.3.1.110) produces a tyramine-derived hydroxycinnamoyl acid amide (e.g., *N-p-*coumaroyltyramine). Agmatine N4-coumaroyltransferase (E.C. 2.3.1.64) produces an agmatine-derived hydroxycinnamic acid amide (e.g., *N*-*p*-coumaroylagmatine).

Hydroxycinnamic acid amides (HCAAs) are obtained from the condensation of a cinnamic acid (or its analogues) with a polyamine or a phenylethylamine (Figure 5) [24,49]. As such, HCAAs are considered polyamine end products, thus allowing their storage and regulation [49]. Corroborating this, a considerable portion of polyamines found in plant cells are either mono-, di-, or trisubstituted with phenolic acids [49]. Aromatic amino acids, agmatine as well as octopamine and the neurotransmitters dopamine and serotonin, can be condensed to form HCAAs [49]. There also are aromatic L-amino acid decarboxylases (E.C. 4.1.1.28) that are not substrate specific and are capable of decarboxylating multiple aromatic amines. These type II decarboxylases use a pyridoxal phosphate molecule (vitamin B_6_) as a cofactor [24]. The enzymes catalyzing the formation of HCAAs belong to the group of BAHD acyltransferases [46,50]. Lignanamides are probably produced by the oxidative phenolic coupling of two HCAAs (e.g., *N*-*p*-coumaroyltyramine, *N*-caffeoyltyramine, etc.), but no studies detailing their biosynthesis in *Cannabis* have been found [29]. This underscores the lack of research efforts in this field, even though a fair number of papers describe their bioactivity in numerous species. In *Cannabis* inflorescences of hemp varieties, the HCAA *N*-caffeoyltyramine is found in levels ranging from 0.1 to 76.2 mg/kg, with an average in the tens of mg/kg for four different cultivars [51]. Cannabisin A, B, and C are found in levels ranging from 0.003 to 2.85 mg/kg from the same samples previously described, with cannabisin A generally found in higher concentrations and cannabisin C in lower ones, with cannabisin B right between the two [51].

### 3.4. Biosynthesis of Spermidine and Spermidine Alkaloids

**Figure 6:**
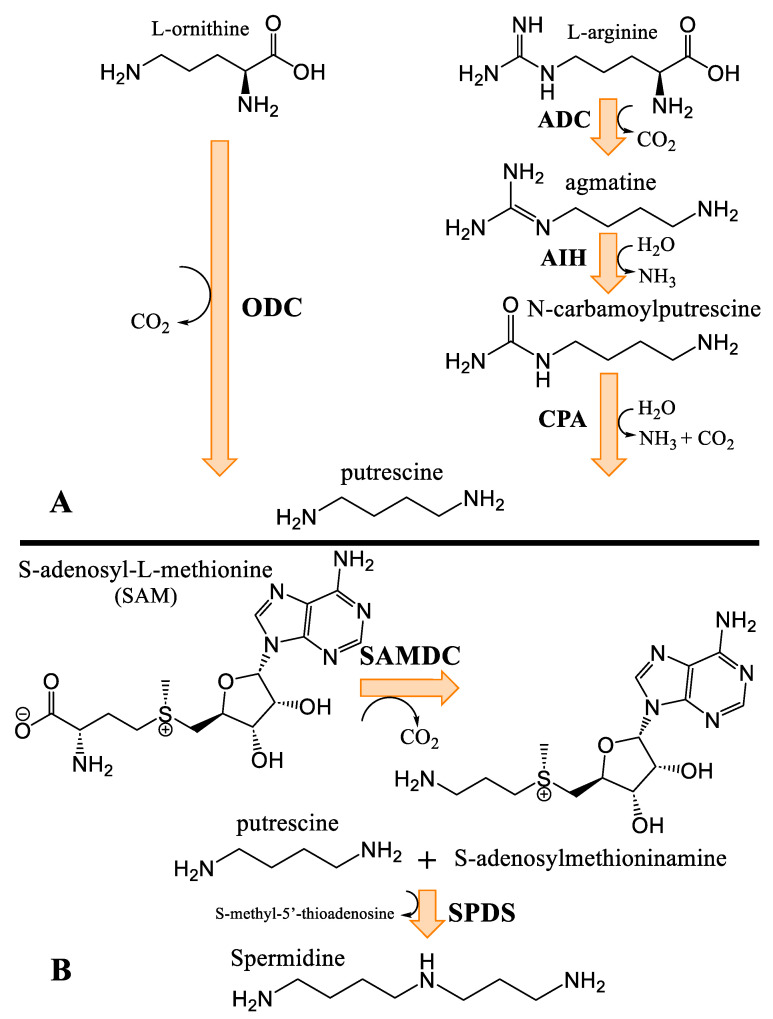
Spermidine biosynthetic pathway. (**A**) General biosynthesis of putrescine. ODC, ornithine decarboxylase. Arrow represents one biochemical reaction. ADC, arginine decarboxylase. AIH, agmatine iminohydrolase. CPA, *N*-carbamoylputrescine amidase. Ornithine decarboxylase (E.C. 4.1.1.17) produces putrescine and CO_2_ from L-ornithine. Arginine decarboxylase (E.C. 4.1.1.19) produces agmatine and CO_2_ from L-arginine. Agmatine iminohydrolase (E.C. 3.5.3.12) produces *N*-carbamoylputrescine and NH_3_ from agmatine. *N*-carbamoylputrescine amidase (E.C. 3.5.1.53) produces putrescine through hydrolysis, generating NH_3_ and CO_2_ as well. (**B**) General biosynthesis of spermidine. SAMDC, *S*-adenosylmethionine decarboxylase. SPDS, spermidine synthase. *S*-adenosylmethionine decarboxylase (E.C. 4.1.1.50) produces *S*-adenosylmethioninamine and CO_2_ through decarboxylation of *S*-adenosyl-L-methionine. Spermidine synthase (E.C. 2.5.1.16) utilizes the aforementioned *S*-adenosylmethioninamine to transfer a propylamine group to putrescine, generating spermidine and *S*-methyl-5′-thioadenosine.

Given their vital function within organisms, the biosynthetic pathways of polyamines are similar from one kingdom of life to another [24]. Polyamine anabolism begins with either the non-proteinogenic amino acid L-ornithine or with the amino acid L-arginine, which are decarboxylated by ornithine decarboxylase (or arginine decarboxylase, respectively) (Figure 6). The production of putrescine from arginine requires two additional steps, compared to the direct ornithine pathway. The biosynthesis of spermidine requires an aminopropyl group donor, obtained through *S*-adenosylmethioninamine.

**Figure 7:**
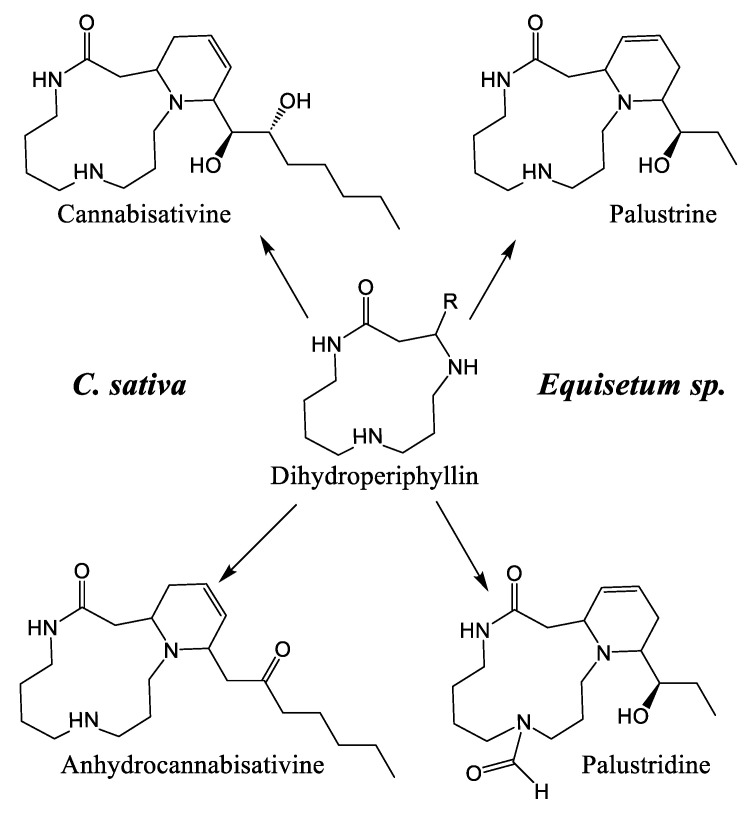
Dihydroperiphyllin skeleton and similar *Cannabis* and *Equisetum* sp. alkaloids.

Cannabisativine was first reported in 1975, and its structure was elucidated by X-ray crystallography [39]. Little is known about its biosynthesis, other than the two terminal nitrogens of spermidine, which are thought to be condensed onto another molecule, most likely a 14-carbon fatty acid, generating a dihydroperiphyllin-type skeleton [29]. Given the immense diversity of natural products, and with a flabbergastingly low number of enzymes, two mechanisms are laid out to explain their advent. The first is that organisms tend to make use of metabolic branches common to other species, forming shared molecular skeletons and founding specialized metabolic pathways where the skeletons are tailored by various enzymes belonging to a specific phylogenetic group (scaffolding principle). The second one is the use by homologous enzymes of substrates that are more or less similar, thus allowing molecular constructions related through the enzyme catalyzing the reaction, but nevertheless molecularly different [52]. In the case of cannabisativine and its dehydration product, anhydrocannabisativine, the second explanation is likely to be the most plausible. Indeed, very similar alkaloids have been reported in several species of the *Equisetum* genus, including palustrine and palustridine, characterized by a 13-atom macrocycle, formed, according to the hypothesis, by the enantioselective condensation of the terminal nitrogens of spermidine, on a 10-carbon fatty acid (Figure 7) [29].

**Figure 8:**
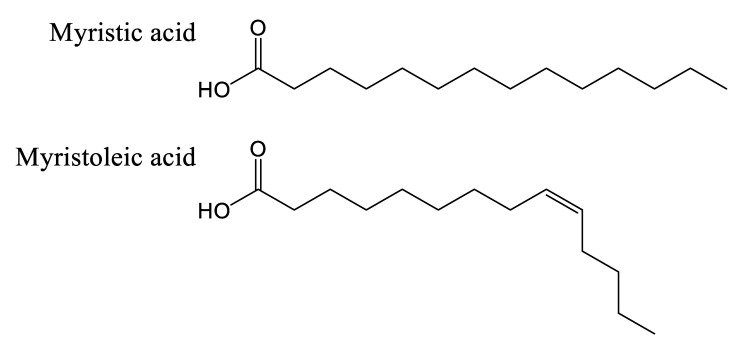
Structure of fatty acids potentially involved in the biosynthesis of spermidine alkaloids of *Cannabis*.

This suggests that a similar enzymatic reaction occurs in *Cannabis*, as in *Equisetum* species, but using a 14-carbon fatty acid. A notable difference between the dihydroperiphyllin alkaloids of *Equisetum* species and *Cannabis* is the position of the double bond in the six-atom heterocycle. Indeed, the double bond is between C5 and C6 of the fatty acid for cannabisativines, as opposed to C4 and C5 for palustrine and palustridine (C1 being the carbon bearing the amide function). Two possibilities then seem conceivable: either the fatty acid has its double bond before the spermidine condensation, and since the fatty acid used is not the same in the two species, this could explain the difference in the position of said double bond; or the condensation takes place with a saturated fatty acid, which is subsequently modified to obtain the desired molecule. However, in extracts of hemp inflorescences, a study reports having identified myristic acid using UHPLC, which is a saturated fatty acid with 14 carbons, representing, depending on the cultivar, between 0.3% and 1.6% of the composition of the fatty acids of the flowers [53]. Additionally, the structure of cannabisativine presents two hydroxyl groups on C8 and C9 of its fatty acid moiety. Although it is possible that a hydratase could convert a double bond to an -OH group, this reaction alone would not be sufficient to produce cannabisativine, requiring an additional hydration. Thus, myristic acid is a realistic candidate for cannabisativine synthesis, which may require cytochrome P450 enzymes to obtain the two hydroxyl groups and quite possibly a dehydrogenase to produce the double bond at C5–C6. The other potential candidate, its close cousin, myristoleic acid, also contains 14 carbons, but is distinguished by its double bond at C9–C10 (Figure 8).

**Figure 9:**
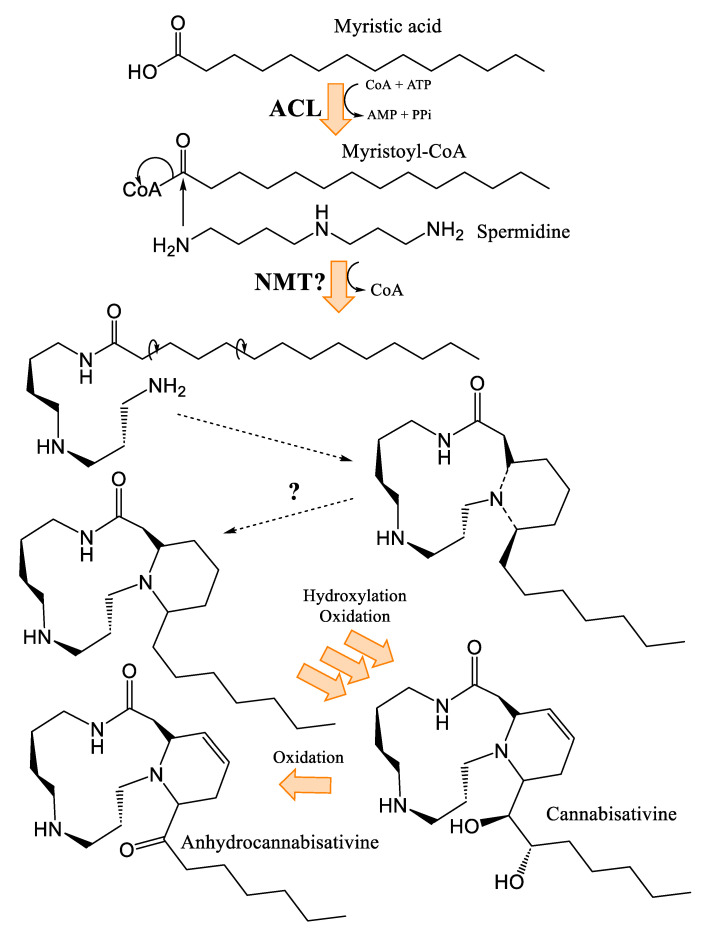
Hypothetical biosynthetic pathway of cannabisativine and anhydrocannabisativine. ACL, acyl-CoA ligase. Arrow represents one biochemical reaction and dotted arrow represents unknown (?) putative reaction. NMT, *N*-myristoyltransferase. First step is thought to be a condensation between spermidine and a fatty acid, presumably myristic acid. The open-end nitrogen atom of spermidine would then be fixed on C-3 and C-7 of the myristoyl moiety. A series of oxidation would generate the end products cannabisativine and anhydrocannabisativine. The order in which those oxidation reactions occur still is unknown.

Furthermore, it is more likely that myristic acid is the precursor of these alkaloids between the two presented candidates, given the ubiquity in eukaryotes of the enzyme *N*-myristoyltransferase (NMT, E.C. 2.3.1.97), catalyzing the condensation of a myristic acid linked to coenzyme A, a product of the reaction of an acyl-CoA ligase (E.C. 6.2.1.3), to the nitrogen atom on the N-terminal glycine of a protein, forming an amide function [54]. This enzymatic reaction is consistent with the hypothesized first step in the biosynthesis of cannabisativine, with the only difference being the conjugation of an amine from spermidine, rather than a terminal glycine of a peptide (Figure 9). Additionally, the double bond of myristoleic acid would restrict freedom of movement, possibly hindering its positioning in the active site, unlike myristic acid. Anhydrocannabisativine should normally follow the same biosynthetic pathway as its analogue. It remains uncertain whether the dehydration of cannabisativine can occur spontaneously or whether it requires the activity of an enzyme. On the other hand, it is known that it is possible to obtain anhydrocannabisativine by sufficiently heating cannabisativine (180–185 °C, 2 min) [40]. Furthermore, cannabisativine seems to be the predominant spermidine alkaloid in *Cannabis* roots, with reported levels of 2.5 mg/kg, compared to anhydrocannabisativine, with levels of 0.3 mg/kg [40,55].

### 3.5. Flavonoids of Cannabis

**Figure 10:**
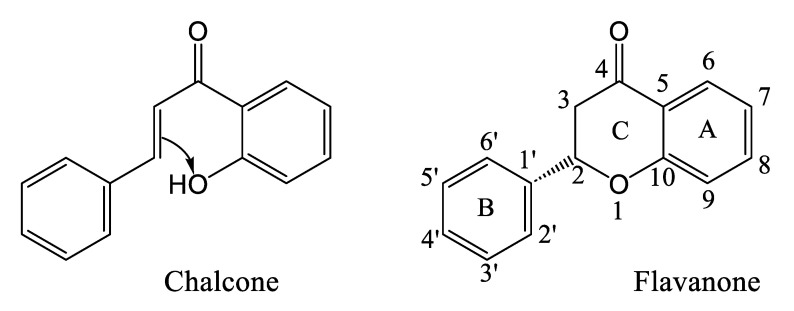
Intramolecular cyclization of chalcones and flavanone numbering.

**Figure 11:**
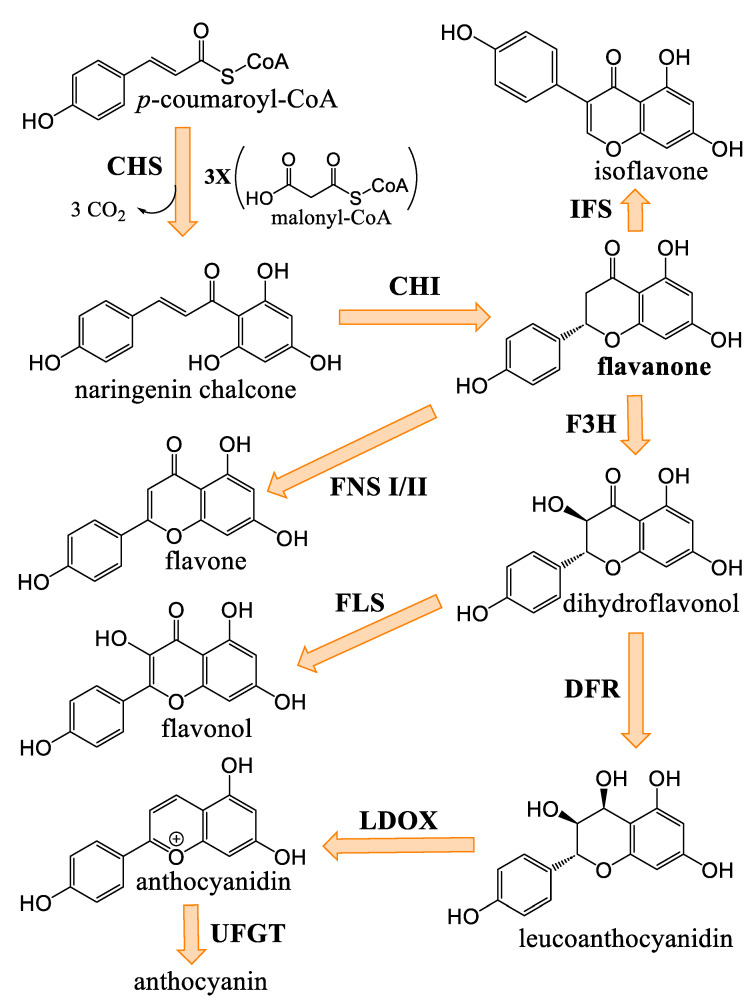
General biosynthetic pathway of flavonoids from *p*-coumaroyl-CoA. Flavanone is in bold, to emphasize its precursor role to other flavonoids. Arrow represents one biochemical reaction. CHS, chalcone synthase. CHI, chalcone isomerase. IFS, isoflavone synthase. FNS, flavone synthase I and II. F3H, flavanone 3-dioxygenase. FLS, flavonol synthase. DFR, dihydroflavonol 4-reductase. LDOX, leucoanthocyanidin dioxygenase. UFGT, UDP-glucose–flavonol O-glucosyltransferase. Chalcone synthase (E.C.2.3.1.74) produces a chalcone from a phenylpropanoid thioester (e.g., *p*-coumaroyl-CoA) by addition and subsequent cyclization of 3 molecules of malonyl-CoA. Chalcone isomerase (E.C.5.5.1.6) produces a flavanone from a chalcone through an intramolecular cyclization of an oxygen of the malonyl portion of the chalcone with the double bond originating from coumaric acid (see Figure 10). Isoflavone synthase (E.C. 1.14.14.87) produces an isoflavone from a flavanone by migration of their aryl group, from C-2 to C-3, combined with an oxidation of C-2, resulting in the double bond formation between C-2 and C-3. Flavone synthase I and II (E.C. 1.14.20.5) (E.C. 1.14.19.76) produce flavones from flavanone by oxidizing C-2 and C-3, forming a double bond. Flavanone 3-dioxygenase (E.C. 1.14.11.9) produces a dihydroflavonol from a flavanone by oxidizing C-3 using oxoglutarate, resulting in a hydroxyl group. Flavonol synthase (E.C. 1.14.20.6) produces a flavonol from a dihydroflavonol by oxoglutarate-dependent oxidation of C-2 and C-3, resulting in a double bond. Dihydroflavonol 4-reductase (E.C. 1.1.1.219) produces a leucoanthocyanidin from a dihydroflavonol by reducing the ketone group to a hydroxyl. Leucoanthocyanidin dioxygenase (E.C. 1.14.20.4) produces anthocyanidin from leucoanthocyanidin by oxidation of the C ring, resulting in loss of hydroxyl groups and positive charge acquisition in the heterocycle. UDP-glucose–flavonol *O*-glucosyltransferase (E.C. 2.4.1.91) produces anthocyanin by addition of sugar moieties to anthocyanidins.

Flavanones are the dominant precursors for the biosynthesis of the different subclasses of flavonoids. It is noteworthy that the intramolecular cyclization of chalcones occurs spontaneously at room temperature, whereas stereospecific cyclization requires the enzyme (Figure 10) [56]. Flavanones are generally modified according to three types of reaction: oxidation, hydroxylation, and displacement of the aryl group (Figure 11) [56]. Naringenin is such a flavanone, which can be transformed into different subclasses of flavonoids in *Cannabis*. Izzo et al. (2020) quantified the molecule in the plant’s inflorescences, with levels neighboring 0.5–1.0 mg/kg [51]. Such a low concentration compared to other flavonoids could be explained by naringenin’s role as an upstream precursor these compounds. Cannflavin A and B were quantified by Allegrone et al. (2017); their concentration in pre-flowering leaves ranges from 21 to 280 mg/kg for cannflavin A and 9 to 106 mg/kg for cannflavin B, with cannflavin A being the most abundant [57]. In *Cannabis* inflorescences of the hemp cultivar, cannflavin A ranges from 20 to 130 mg/kg, and cannflavin B from 12 to 215 mg/kg, with cannflavin B being the most abundant [51]. It is unknown if this seeming discrepancy in cannflavin levels is due to the cultivar, stage of development, tissue, environmental growth conditions, or something else.

Flavone synthase I is a dioxygenase requiring a molecule of 2-oxoglutarate to function, while FNS II is an enzyme of the cytochrome P450 family [56]. Flavones in *Cannabis* are namely exemplified by luteolin and apigenin. Luteolin seems to be produced in higher concentrations than apigenin across multiple cultivars. Indeed, mean levels of luteolin are reaching 20 mg/kg depending on the cultivar, with lower ones reaching 10 mg/kg. Apigenin, on the other hand, has mean levels of around 6 mg/kg [51]. 7-*O*-glycosylated forms of those flavones have been identified, with levels comparable to luteolin and apigenin. Flavanone 3-dioxygenase is important in the regulation of flavonoid metabolism, concomitant with flavone synthases I and II [56,58]. Dihydroflavonols serve as precursors for the biosynthesis of anthocyanins and flavonols [58]. Catechin and epicatechin are dihydroflavonols found in *Cannabis,* with levels ranging from 0.1 to 334.0 mg/kg, with a mean of around 50 mg/kg for catechin and ranging 1.6 to 194.6 mg/kg with a mean of around 60 mg/kg for epicatechin [51]. Flavonols quercetin and kaempferol are, respectively, found in ranges of 6.2–58.5 mg/kg, with a mean of around 16 mg/kg, and 0.3–13.6 mg/kg, with a mean around 5 mg/kg.

#### Biosynthesis of Cannflavins

**Figure 12:**
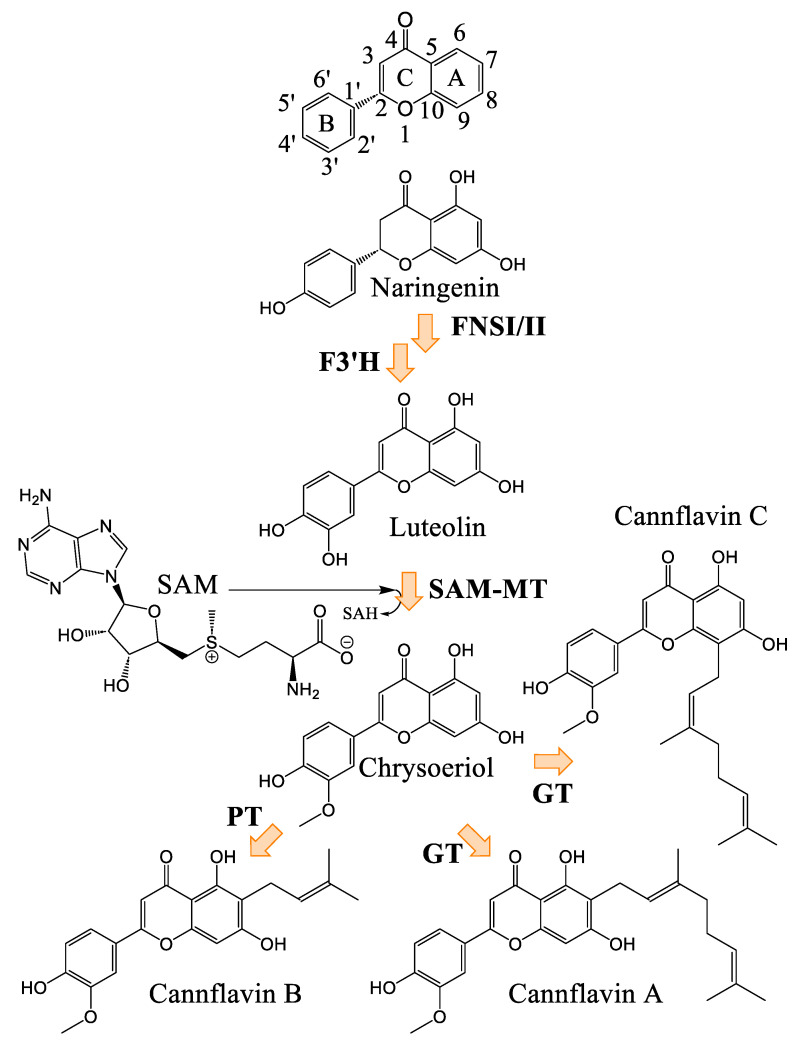
Biosynthesis of cannflavins. FNSI/II, flavone synthase I and II. F3′H, flavonoid 3′-hydroxylase. Arrow represents one biochemical reaction. SAM-MT, S-adenosyl-L-methionine methyltransferase. GT, geranyltransferase. PT, prenyltransferase. SAH, S-adenosyl-L-homocysteine. Luteolin can be obtained from naringinin by the consecutive action of a flavone synthase (I or II) (E.C. 1.14.20.5) (E.C. 1.14.19.76), which will produce a double bond at C2 and C3, and a NADPH-dependent flavonoid 3′-hydroxylase (E.C. 1.14.14.82). A SAM-methyltransferase will methylate the hydroxyl group just added in 3′ of the B ring by the F3′H to form chrysoeriol and produce SAH. A geranyltransferase and a prenyltransferase will add their respective group on the C7 of the A ring of chrysoeriol, to obtain cannflavins A and B, respectively. To produce cannflavin C, a geranyltransferase adds its geranyl group to C-9 of the A ring of chrysoeriol.

*Cannabis* flavonoids are found primarily in the leaves and flowers, but have also been isolated and identified from the pollen and stem of the plant [12]. There is no evidence to date for the presence of flavonoids in *Cannabis* roots. *Cannabis* seeds are thought not to contain prenylflavonoids, but germination induces the production of cannflavin A and B in leaves of plants that are not yet producing cannabinoids. Flavonoid concentrations decrease as the plant grows [12]. Flavonoid biosynthesis has only been slightly investigated in *Cannabis*. The biosynthetic pathways are well described, but several enzymes remain to be confirmed in planta. However, it is likely that the biosynthetic pathway is closely related to that of plants already studied, since three flavones widespread in the plant kingdom are found in *Cannabis*, namely apigenin, luteolin, and chrysoeriol, and their glycosylated analogues [59]. The proposed pathway in *Cannabis* would therefore correspond to that presented in Figure 11. The biosynthesis of cannflavins probably occurs from the flavone luteolin (Figure 12). Rea et al. provided new evidence in 2019 that suggests that the sequential order of cannflavin biosynthesis would be as follows: 3′-methylation of luteolin, followed by prenylation. Indeed, they identified, by phylogeny, a candidate enzyme for the prenylation reaction (*Cs*PT3) and a candidate for the methylation reaction (CsOMT21) of luteolin. The candidate for prenylation accommodates both dimethylallyl pyrophosphate (DMAPP) and geranyl pyrophosphate (GPP), with chrysoeriol as the preferred substrate, rather than luteolin. Furthermore, the fact that no reports of prenylated luteolin have been produced suggests that the first of the two enzymatic reactions is methylation, generating chrysoeriol [59]. The candidate enzyme CsOMT21 does not methylate flavonones lacking a 3′-hydroxyl group, suggesting specificity for this position. Additionally, the preferred substrate of this enzyme is luteolin, but it also methylates quercetin (a flavonol) at the 3′ position, with 57% of the activity of luteolin. Cannflavin A and B have been quantified in some hemp cultivars and show comparable levels. They are found in the range of 19.6–130.0 mg/kg and 11.9–215.5 mg/kg, with a mean of around 62 and 84 mg/kg, respectively.

### 3.6. Stilbenes of Cannabis

**Figure 13:**
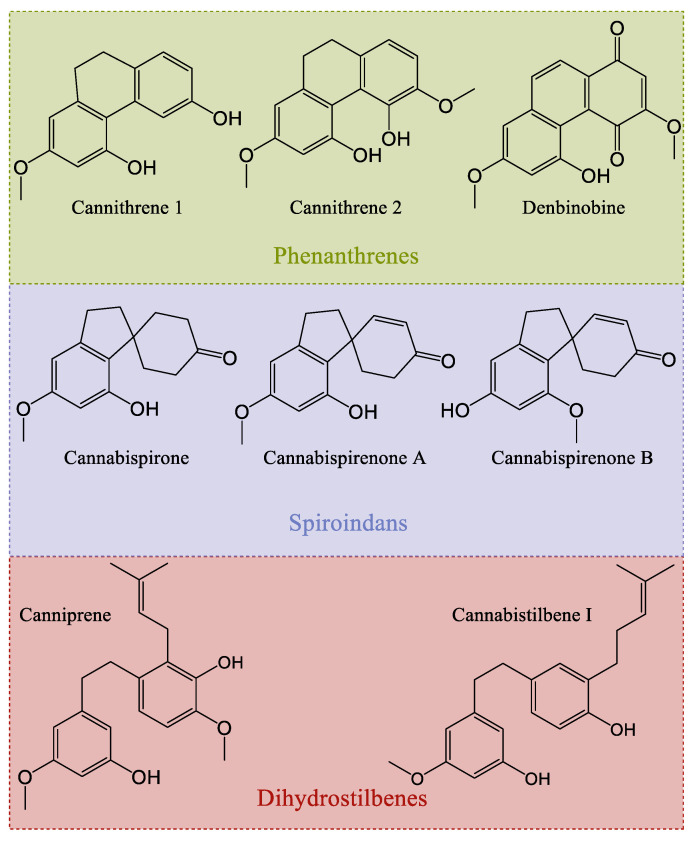
Classification of stilbenes identified in *Cannabis* according to their chemical structure.

As it relates to *Cannabis*, stilbenes are a group of phenolic compounds that can be distinguished into three different classes, based on their chemical structure: phenanthrenes, dihydrostilbenes, and spiroindans [60] (Figure 13). To date, a total of thirty-eight stilbenes have been identified: seven phenanthrenes, fifteen dihydrostilbenes, and sixteen spiroindans [5,12,19]. Like flavonoids, stilbenes are found in the flowers, leaves, and stems of the *Cannabis* plant, and they share the same biosynthetic origin. No mention of stilbenes in the roots of *Cannabis* has been reported. Stilbene synthase (STS) is thought to have evolved independently from chalcone synthase several times during natural history (75–90% sequence homology), explaining the disparate distribution of stilbenes in the plant kingdom, while flavonoids are ubiquitous [35,61]. Some researchers have hypothesized that stilbene production could compete with flavonoid production in vivo in *Cannabis* [60]. Indeed, there appears to be an inverse relationship between stilbene and flavonoid concentrations in *Cannabis* cultivars, suggesting competition at the level of their precursors, cinnamic acids [57]. One study investigated a *Cannabis* type II 4-coumarate ligase (*Cs*4CL4) and a bibenzyl synthase (*Cs*BBS2), suspected to be involved in stilbene production, which have a higher affinity for *p*-coumaric and caffeic acids among other cinnamic acids, corroborated by the chemical structures of stilbenes reported in *Cannabis* [57,62].

#### Biosynthesis of Cannabis Stilbenes

**Figure 14:**
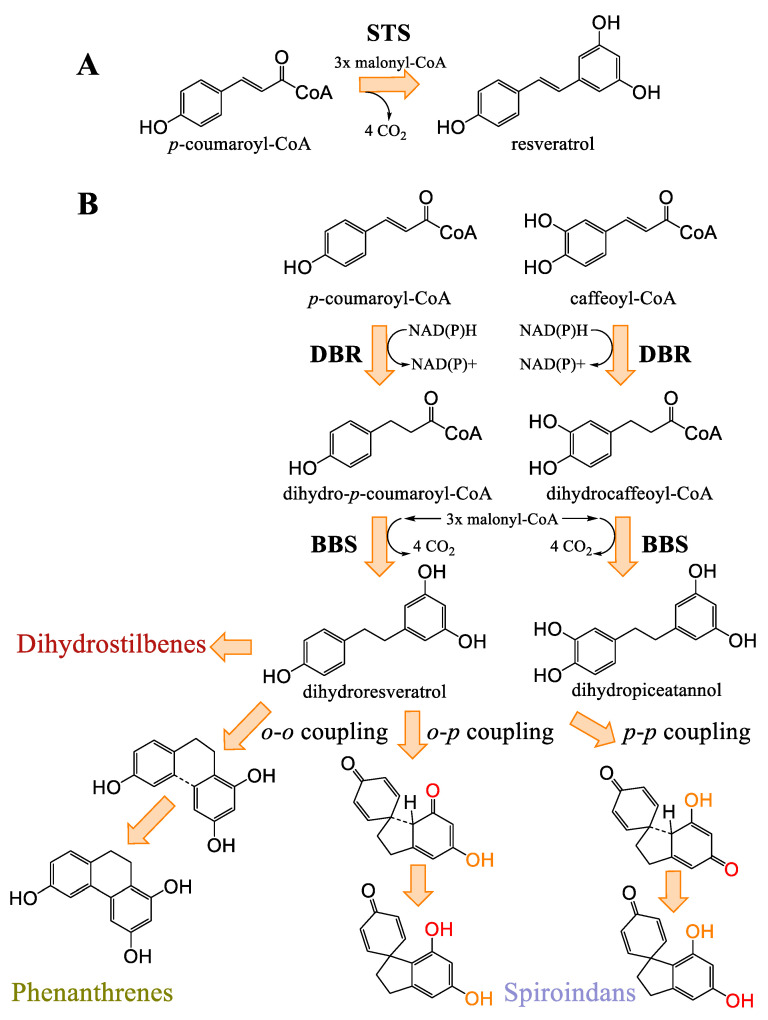
General biosynthesis pathway of *Cannabis* stilbenes. (**A**) Enzymatic reaction for the production of resveratrol. Arrow represents one biochemical reaction. STS, stilbene synthase. Stilbene synthase (2.3.1.95) produces resveratrol and CO_2_ from *p*-coumaroyl-CoA by subsequent addition of three malonyl-CoA molecules, and their cyclization, releasing four CO_2_ molecules. (**B**) Enzymatic reactions carried out in *Cannabis* for the production of stilbenes. DBR, double bond reductase. BBS, bibenzyl synthase. Note that the resulting structures of the oxidative coupling reactions (i.e., phenanthrenes and spiroindans) were conceived using the structure of dihydroresveratrol for simplicity. Double bond reductase (E.C. 1.3.1.117) produces dihydro-*p*-coumaroyl-CoA from *p*-coumaroyl-CoA and dihydrocaffeoyl-CoA from caffeoyl-CoA through NAD(P)H oxidation. BBS (E.C. 1.3.1.74) produces dihydroresveratrol from dihydro-*p*-coumaroyl-CoA and dihydropiceatannol from dihydrocaffeoyl-CoA, through addition and cyclization of three subsequent malonyl-CoA molecules, releasing four CO_2_ molecules. From dihydrostilbenes, different oxidative coupling reactions can take place, producing phenanthrenes (*o*-*o* coupling) and spiroindans (*o*-*p* and *p*-*p* coupling).

In a general sense, stilbenes can be biosynthesized via one of two pathways: either following the catalytic activity of STS or of BBS (Figure 14). These two enzymes essentially accommodate the same precursors, namely cinnamic acid thioesters, particularly *p*-coumaric and caffeic acids for STS and their dihydrated equivalents for BBS [62]. *Cannabis* most probably uses BBS rather than STS. A structural study of the *Cannabis* bibenzyl synthase 2 enzyme (*Cs*BBS2) indicates that it has undergone a narrowing at the level of the hydrophobic pocket of the active site, when compared to STS, explaining the greater affinity for the dihydrated form of cinnamic acid thioesters [62]. It is useful to note that ferulic acid and cinnamic acid (and their dihydrated homologues) can be accommodated by these enzymes, but significantly less well than their preferred substrates, while sinapic acid is not viable at all as a precursor for these enzymes [62]. This would mean that stilbenes presenting methoxy groups in their structure are likely to have had them added following the production of the stilbene/bibenzyl skeleton by methyltransferases, as is the case for flavonoids, rather than obtaining these groups from precursors such as ferulic or sinapic acid. Boddington et al. identified nine *C. sativa* double bond reductase homologues (*Cs*DBR1-9) in *Cannabis*, and all are classified as NAD(P)H-dependent alkenal/one reductases. They determined that the enzymes *Cs*DBR2 and *Cs*DBR3 catalyze the reduction reaction of *p*-coumaroyl-CoA to dihydro-*p*-coumaroyl-CoA, and caffeoyl-CoA to dihydrocaffeoyl-CoA, respectively. Their results of enzymatic tests on the enzymes *Cs*BBS1 and *Cs*BBS2, suspected of operating the biosynthesis of bibenzyls in *Cannabis*, indicate that the enzyme *Cs*BBS2 accommodates the two substrates, dihydro-*p*-coumaroyl-CoA and dihydrocaffeoyl-CoA, while *Cs*BBS1 is incapable of doing so [62].

## 4. Biological Activity of Cannabis Metabolites

### 4.1. Polyamines and Their Physiological Roles

**Figure 15:**
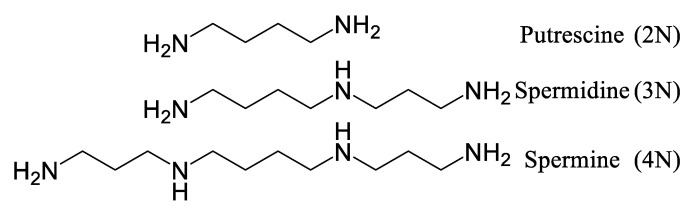
Structure of some polyamines from the three different types: diamines, triamines, and tetraamines.

As mentioned in Section 2, polyamines (Figure 15) play a fundamental role in living organisms’ ability to adapt to stresses, notably by interacting with macromolecules. These stabilizing interactions may play a protective role during periods of stress, as suggested by a study, where DNA was protected from heat-induced depurination in vitro, where 1 mM of spermine reduced depurination by 50%. Furthermore, it was observed that the higher the number of nitrogen atoms in the tested polyamines, the better the protection from heat [25]. Polyamines will also influence, through their interaction with chromatin, the sites of the genome that are accessible to DNA/RNA polymerase. Some kinases have also been reported to be impacted at the transcriptional and post-translational levels by polyamines [63]. Polyamines are crucial for development, but also in the adaptive response to different stresses. Their catabolism generates reactive oxygen species (ROS), but in their free (unconjugated) form, polyamines also serve as ROS-scavenging molecules, thus allowing a meticulous regulation of their abundance and availability [24,46,50].

### 4.2. Biological Activity of Cannabis Alkaloids and Other Nitrogen-Containing Compounds

No study yet published has delved into the biological activity of isolated spermidine alkaloids in *Cannabis*. Thus, their potential activity is still unknown. However, it can be assumed that they do possess some. Indeed, as described above, spermidine is an important component fulfilling many crucial roles at the cellular level and has a notable impact on cell growth. Even if only by catabolism, cannabisativine has a spermidine moiety in its structure, and it could then be degraded to release the polyamine when cellular needs require it. It is interesting to think that if cannabisativine has a biological activity, it would be one intimately linked to its polyamine portion, which may be possibly useful for countering certain specific stresses. The other nitrogenous compounds of *Cannabis* have varied activities. Hordenine is a fairly decent prospect in antibiotic therapy, inhibiting some gram-negative bacteria, their biofilm, and quorum-sensing abilities, and is a promising anti-inflammatory compound for multiple conditions, notably neurodegenerative diseases like Parkinson’s and Alzheimer’s. It also demonstrates some potential to alleviate muscular hypotrophy. HCAAs found in *Cannabis*, i.e., N-trans-*p*-coumaroyltyramine, N-trans-feruloyltyramine, and N-trans-caffeoyltyramine, possess antiproliferative, anti-inflammatory, antineoplastic, neuroprotective, antioxidant, antimicrobial, anticholinesterase, anti-diabetic, antifeeding, and slight analgesic activities [29]. As lignanamides are dimers of HCAAs, they share many biological activities, namely anti-inflammatory, antiproliferative, antioxidant, anti-tyrosinase, and anticholinesterase activities. Some lignanamides show particular promise in neuroinflammatory appeasement. The antioxidant activity of cannabisins is also of importance.

#### 4.2.1. In Vitro Activity of Cannabis Nitrogen-Containing Compounds

Hordenine from barley (Hordeum vulgare) is reported to have a repellent activity towards grasshoppers [29]. It also possesses antibacterial activity, with a minimal inhibitory concentration (MIC) of 2.5 mg/mL against the Pseudomonas aeruginosa PAO1 strain [64]. Zhou et al. (2018) also tested the quorum-sensing (QS) inhibitory activity of hordenine [64]. Acylated homoserine lactones (AHLs) are gram-negative autoinducers necessary for biofilm formation. Two notable AHLs were confirmed extracellularly by LC-MS/MS from *P. aeruginosa* PAO1: N-(3 oxododecanoyl)-L-homoserine lactone (3-oxo-C12-HSL) and N-butanoyl-L-homoserine lactone (C4-HSL). Thus, Zhou et al. (2018) tested the effect of hordenine exposure during 24 h in the culture medium, with concentrations of 0.5, 0.75, and 1.0 mg/mL [64]. They observed a reduction of 69%, 74%, and 79% for C4-HSL, with respect to the listed concentrations, when compared to control. They noted a reduction ranging from 24% to 66% for 3-oxo-C12-HSL with the same hordenine concentrations, suggesting interference by hordenine in AHL production. Additionally, they reported hordenine’s ability to inhibit biofilm formation in the PAO1 strain, with concentrations lower than 1.0 mg/kg, as well as significantly enhance the efficacy of the antibiotic netilmicin in biofilm formation inhibition [64]. Hordenine is also capable of disrupting and thinning preformed biofilms, especially when combined with netilmicin, and suppressing virulence factors of *P. aeruginosa*, namely protease and elastase activity, pyocyanin and pyoverdine production, and rhamnolipid and alginate activities, even downregulating QS-related genes *lasI, lasR, rhlI*, and *rhlR* by nearly 50% [64]. Similarly, hordenine’s quorum-sensing inhibitory activity was tested against the *Serratia marcescens* NJ01 strain by Zhou et al. (2019) [65]. They reported an MIC of 2.5 mg/mL for *S. marcescens*, which is the same as *P. aeruginosa* PAO1, and noted that concentrations of 25, 50, and 100 μg/mL of hordenine in an LB broth medium for 24 h resulted in a diminution of C4-HSL levels of 40%, 60%, and 80%, respectively [65]. Withholding details, they found results resembling those obtained with *P. aeruginosa* PAO1, with hordenine reducing biofilm autoinducer levels, inhibiting biofilm formation, lessening virulence factors, altering preformed biofilm architecture (resulting in higher antibiotic sensitivity with ciprofloxacin), and downregulating QS-related genes. Chikazawa and Sato (2018) investigated hordenine’s β_2_-adrenergic receptor (β_2_-AR) agonistic capabilities in HEK293 cells expressing either human or mouse β_2_-AR, using a luciferase assay [66]. After 6 h of hordenine exposure, the cells were tested for luciferase activity. They found hordenine to be an agonist of mouse β_2_-AR in a dose-dependent manner, with little to no effect with 10 μM, a twofold increase in luciferase activity compared to control with 30 μM of hordenine, and a significant more than sevenfold increase at 100 μM of hordenine. Regarding HEK293 cells expressing human β_2_-AR, hordenine at 100 μM was also confirmed as an agonist of the receptor.

##### HCAA Activity In Vitro

Synthesized N-trans-coumaroyltyramine was tested for antiproliferative activity against Jurkat and U937 human cell lines. Cells were treated with concentrations of 30, 60, and 90 μM of N-trans-coumaroyltyramine for 18 h, and total cell death was measured by the trypan blue exclusion method and a nonradioactive cell proliferation method by Park and Schoene (2002) [67]. They reported a notable decrease in the number of living cells with 30 μM of N-trans-coumaroyltyramine (40% for U937, 32% for Jurkat), with increasing efficacy at higher concentrations. Also, they brought attention to the fact that numerous U937 and Jurkat cells treated with the compound, especially at doses higher than 50 μM, halted predominantly in the S phase of the cell cycle. They then tested the effect of this HCAA on the epidermal growth factor receptor (EGFR), a tyrosine kinase that is overexpressed in certain cancer types. They observed a dose-dependent inhibition of EGFR by N-trans-coumaroyltyramine, with a 20% reduction in activity with 30 μM in the U937 cell line, and a 75% reduction in activity with 120 μM. Comparable results were produced for the Jurkat cell line. These results suggest that N-trans-coumaroyltyramine interferes with tyrosine kinase activity and its signaling process, necessary in cancerous cell replication, and could induce an apoptotic response. Its antiproliferative activity was tested on HeLa (cervical cancer) and A431 (epidermoid carcinoma) cell lines and it was found that N-p-trans-coumaroyltyramine significantly reduced cell viability in a dose- and time-dependent manner, at 10 μM for a 24 h treatment, and at 5 μM for 48 h of exposure [68]. N-p-trans-coumaroyltyramine was shown to enhance the sensitivity of indomethacin and diclofenac, two nonsteroidal anti-inflammatory drugs with antiproliferative activity against certain cancer types, in MCF-7 (breast cancer) and mitoxantrone-resistant MCF-7 (MCF-7/MX) cell lines. Exposure to 100 μM of N-p-trans-coumaroyltyramine for 48 h decreased MCF-7 viability by more than twofold when combined with 10 μM of both indomethacin and diclofenac [69]. For MCF-7/MX cells, 100 μM of N-p-trans-coumaroyltyramine decreased cell viability by twofold for 10 μM of indomethacin and 1.5-fold for diclofenac. Furthermore, 100 μM of N-p-trans-coumaroyltyramine increased the cytotoxic activity of endoplasmic reticulum stressors, such as thapsigargin (0.0125 μM), tunicamycin (0.3 μM), rotenone (1.0 μM), and hydrogen peroxide (10 μM) in both MCF-7 cell lines [69]. N-trans-feruloyltyramine extracted from laba garlic was reported to possess antiproliferative activity against HepG2 cells, with an IC_50_ = 194 μM, whereas the positive control taxol had an IC_50_ = 26 μM [70]. N-trans-feruloyltyramine and N-trans-caffeoyltyramine exhibited antiproliferative activity against A2780 (ovarian cancer) and Panc1 (pancreatic cancer) cell lines, with feruloyltyramine having an IC_50_ = 9.1 μM (A2780) and IC_50_ = 3.4 μM (Panc1), and caffeoyltyramine having an IC_50_ = 11.2 μM (A2780) and IC_50_ = 7.2 μM (Panc1) [71].

N-trans-coumaroyltyramine was reported to possess moderate acetylcholinesterase inhibitory activity, with an IC_50_ of 48.8 μM [72]. The potential of lignanamides in Alzheimer’s disease treatment was tested by verifying its effect on BACE 1, PPARγ, and PGC-1α expression in amyloid β precursor protein (APP)-producing N2a cells. The BACE 1 enzyme produces monomeric forms of amyloid β peptides, which accumulate in the brain, and is associated with a worsening of the symptoms of the disease. BACE 1 is activated by proinflammatory cytokines, namely IL-1β, IL-6, and TNF-α. N-trans-caffeoyltyramine very significantly reduced the BACE 1 expression of N2a cells at 0.03 μM, more so at 24 h of exposure than 48 h [73].

Additionally, PPARγ, a BACE 1 regulator whose activation inhibits BACE 1, was upregulated by more than twofold with 0.03 μM of N-trans-caffeoyltyramine after 24 h exposure, with a slight decrease after 48 h (just less than twofold), and with 0.08 μM of N-trans-caffeoyltyramine, PPARγ was upregulated more than threefold after 24 h, also with a slight decrease after 48 h (just about threefold). PGC-1α is a coactivator participating in PPARγ transcription and decreased expression is thought to be linked to Alzheimer’s disease [73]. A total of 0.03 and 0.08 μM of N-trans-caffeoyltyramine had comparable effects on PGC-1α expression, upregulating by approximately twofold after 24 h, again with a slight diminution after 48 h of exposure. Furthermore, N-trans-caffeoyltyramine and N-trans-feruloyltyramine extracted from *Bassia indica* and *Agathophora alopecuroides* exhibited the good inhibition of factors implicated in Alzheimer’s disease, namely BACE1 and MAO-B activity, Aβ aggregation, and Tau-protein phosphorylation. N-trans-caffeoyltyramine had an IC_50_ of 10.2 μg/mL (BACE1), 2.6 μg/mL (MAO-B), 6.0 μg/mL (Aβ aggregation), and 6.1 μg/mL (Tau protein), whilst N-trans-feruloyltyramine had, respectively, 11.6 μg/mL (BACE1), 2.2 μg/mL (MAO-B), 1.2 μg/mL (Aβ aggregation), and 2.1 μg/mL (Tau protein) [74]. N-trans-caffeoyltyramine extracted from *Lycium chinense* was reportedly effective in protecting PC12 cells from apoptotic cell death induced by H_2_O_2_ [75]. Where control PC12 cells had an apoptotic rate of 2.7%, the H_2_O_2_ control had a rate of 23.8%. Cells pretreated with 20 μM and 40 μM of N-trans-caffeoyltyramine had an apoptotic rate of 11.7% and 7.3%, respectively, suggesting a neuroprotective function.

In addition to the neuroprotective effects, HCAAs possess antioxidant activity, which seems to be related with the groups in position 3 and 4 of the benzene ring of the cinnamic acid moiety. Free hydroxyl groups in those positions are thought to be key to the enhanced antioxidant effect. Indeed, N-caffeoyltyramine has the highest antioxidant capacity among them in a DPPH-radical scavenging assay, followed by N-feruloyltyramine, N-sinapoyltyramine, and N-p-coumaroyltyramine [76]. Another DPPH-radical assay performed on a Celtis occidentalis extract by Ayanlowo et al. in 2020 corroborates these findings. They reported the antioxidant activity of HCAAs as such: N-trans-caffeoyltyramine (IC_50_ = 31.6 μM) > N-trans-feruloyltyramine (IC_50_ = 47.9 μM) > N-*p*-trans-coumaroyltyramine (IC_50_ = 1378 μM) [77]. Although it is worth considering that the literature data seem to trend in the same general direction regarding HCAA antioxidant activity, published results sometimes have discrepancies in them, so much so that it brought some to hypothesize that plant sources might have an influence on their antioxidant capabilities [78].

N-trans-caffeoyltyramine exhibited anti-inflammatory activity through the inhibition of TNF-α-induced NF-κB production in HEK293/NF-κB-luciferase cells [79]. At 30 μM, N-trans-caffeoyltyramine inhibited NF-κB production by 74% (IC_50_ = 18.4 μM). N-trans-feruloyltyramine, and to a lesser extent N-p-trans-coumaroyltyramine, extracted from Allium hookeri, showed senomorphic activity, inhibiting a senescence-associated secretory phenotype (SASP) [80]. Additionally, they exhibited anti-inflammatory activity, as they decreased IL-1α and IL-8 mRNA in replicative senescent HDF cells at 10 μM. This may suggest that N-trans-feruloyltyramine is a good candidate for fighting age- and senescence-related illnesses. N-trans-feruloyltyramine and N-trans-caffeoyltyramine possess affinity for hepatic nuclear factor 4α (HNF-4α), especially N-trans-caffeoyltyramine, acting as agonists [81]. The activation of HNF-4α induces lipophagy, which results in a release of lipids in hepatic tissue, which can alleviate non-alcoholic fatty liver disease (NAFLD). Also, Lee et al. (2021) brought evidence that 20 μM of N-trans-caffeoyltyramine increased mRNA levels of insulin and HNF-4α by twentyfold and fivefold, respectively; as for N-trans-feruloyltyramine (20 μM), mRNA levels increased more than tenfold for insulin and twofold for HNF-4α. These results suggest that both HCAAs could be beneficial prospects in type II diabetes and hepatic fat regulation.

The antifungal activity of N-trans-caffeoyltyramine extracted from *Lycium chinense* was evaluated by Lee et al. (2004). They reported a MIC of 5 μg/mL against *Candida albincans*, 10 μg/mL against *Saccharomyces cerevisiae,* and 5–10 μg/mL against *Trichosporon beigelii*, compared to the positive control amphotericin B, which had an MIC of 0.63 μg/mL, 5 μg/mL, and 1.25 μg/mL, with respect to each fungus. N-p-trans-coumaroyltyramine exhibited antimicrobial activity against *Escherichia coli*, where the reported IC_50_ was 0.8 μg/mL, compared to the positive control ciprofloxacin (IC_50_ = 0.01 μg/mL) [82]. N-*p*-trans-coumaroyltyramine’s antimicrobial activity could possibly extend to urease-positive bacteria like *Helicobacter pylori*, because of its reported anti-urease activity (IC_50_ = 45.2 μM) against the *Canavalia ensiformis* (jack bean) urease enzyme [83]. N-*p*-trans-coumaroyltyramine and N-trans-feruloyltyramine were reportedly produced in response to *Chilo suppressalis* exposure in rice (*Orza sativa*, cv. Oochikara), suggesting a role in herbivore insect management [84]. The presence of these compounds in the feed given to *C. suppressalis* resulted in a weight loss at concentrations of 100 μg/g, suggesting anti-insect and antifeedant activity. N-*p*-trans-coumaroyltyramine, N-trans-feruloyltyramine, and N-trans-caffeoyltyramine exhibited anti-melanogenesis activity at 50 μM, reducing the amount of melanin produced by alpha melanocyte stimulating hormone (α-MSH) activation in B16F10 melanoma cells [85]. Anti-melanogenesis activity is ranked as follows: N-trans-caffeoyltyramine (IC_50_ = 0.8 μM), N-*p*-trans-coumaroyltyramine (IC_50_ = 6.3 μM), and N-trans-feruloyltyramine (IC_50_ = 20.2 μM). It is worth noting that N-*p*-trans-coumaroyltyramine at 50 μM decreased B16F10 cell viability by more than 50% [85]. All three cinnamamides also exhibited anti-tyrosinase activity.

##### Lignanamide Activity In Vitro

Cannabisin G extracted from *Berberis vulgaris* was reported to possess strong antioxidant activity, with an IC_50_ = 2.7 μg/mL in a DPPH radical-scavenging assay [86]. Cannabisin B extracted from *C. sativa* hemp seed hulls would also possess decent antioxidant properties, with a reported IC_50_ = 11.2 μg/mL in a DPPH radical-scavenging assay, slightly lower than N-*p*-trans-caffeoyltyramine (IC_50_ = 9.42 μg/mL) [87]. In another study concerning hemp seed hulls, cannabisin B demonstrates antiproliferative activity in HepG2 (liver cancer) cells through the inhibition of the AKT/mTOR pathway and autophagy, at concentrations exceeding 167 μM. Furthermore, HepG2 cells treated with cannabisin B tended to arrest the cell cycle in the S phase in a dose-dependent manner [88]. Lignanamides extracted from hemp seeds were tested by Yan et al. (2015) for their antioxidant and anticholinesterase abilities. They found that cannabisin A and D were among the most effective lignanamides tested in DPPH (IC_50_ = 32.9 μM and 23.9 μM), ABTS (IC_50_ = 6.6 μM and 0.5 μM), and ORAC (IC_50_ = 7.3 μM and 73.0 μM) assays, against the positive control quercetin (IC_50_ = 25.5 μM, 0.4 μM, and 9.2 μM) [41]. *C. sativa* lignanamides presenting anticholinesterase activity are 3,3′-demethyl-heliotropamide (IC_50_ = 46.2 μM) and 3,3′ demethyl-grossamide (IC_50_ = 38.7 μM), against the positive control galanthamine (IC_50_ = 2.8 μM). Cannabisin F reportedly exhibited anti-inflammatory activity against BV2 microglia cells. The pretreatment of BV2 cells with doses of 15 μM of cannabisin F significantly reduced IL-6 and TNF-α production induced by LPS (100 ng/mL), at both protein and mRNA levels, and in a dose-dependent manner [89]. Wang and colleagues also provided evidence that the anti-inflammatory activity of cannabisin F is SIRT1-dependent, and acts by inhibiting NF-κB signaling. Grossamide also reduced pro-inflammatory cytokines IL-6 and TNF-α levels of BV2 cells treated with LPS (100 ng/mL, 24 h), with concentrations as low as 10 μM [90]. However, 20 μM of grossamide was necessary to significantly diminish both cytokines’ mRNA levels. In the same fashion as cannabisin F, grossamide mediates the NF-κB signaling pathway, but through the lessening of MyD88 and TLR4 expression, as demonstrated by Luo and colleagues. Cannabisin F from *Solanum nigrum* was tested for its neuroprotective activity against SH-SY5Y cells to evaluate its potential in neurodegenerative disorder treatment. Li et al. (2019) provided evidence that cannabisin F (12.5 μM) reduced MPP^+^-induced apoptosis in SH-SY5Y cells, as well as increased cellular autophagy, resulting in neuroprotective effects [91]. Peripheral blood mononuclear cells (PBMCs) inflamed with 10 μg/mL of LPS and treated with 100 μM of cannabisin D extracted from *Dracaena usambarensis* had a 70.5% reduction in IL-1β levels and an 88.7% reduction in TNF-α levels [92]. Grossamide reduced IL-1β and TNF-α levels by 97.9% and 99.9%, respectively, as well as IL-2 and GM-CSF levels by 88.4% and 98.4%, respectively, exceeding anti-inflammatory effects of the positive control ibuprofen. Cannabisin A and B, isolated from hemp seed husks, were reported as tyrosinase inhibitors, with an IC_50_ of 3.3 μM and 8.1 μM, respectively [93]. Kim et al. confirmed that cannabisin A lowered the melanin content in α-MSH-induced B16F10 cells (melanoma) when cells were treated with 1 μM of the compound for 24 h. The anti-tyrosinase activity of cannabisin A acts in a dose-dependent manner.

#### 4.2.2. In Vivo Activity of Cannabis Nitrogen-Containing Compounds

Hordenine was tested against an ulcerative colitis mouse model by Xu et al. in 2023. Mice were given a 4% dextran sodium sulphate (DSS) solution during the first 7 days to induce ulcerative colitis and hordenine-treated mice received 12.5 mg/kg, 25 mg/kg, or 50 mg/kg from day 1 to 12 [94]. Mice were harvested on day 13. They found that mice treated with 50 mg/kg of hordenine had a diminution in production of IL-6, IL-1β, and TNF-α. Furthermore, the anti-inflammatory activity of hordenine was observed through the inhibition of expression of SPHK1, S1PR1, Rac1, and p-STAT3. As well, the enhanced expression of tight junction proteins ZO-1 and occludin suggest a role in colon epithelial cell healing [94]. Hordenine was reported as a β_2_-AR agonist in mice skeletal muscle by Chikazawa and Sato in 2018. Hordenine (20 mg/kg) diluted in 10% DMSO in water was injected directly in mice quadriceps three times, and RNA of quadricep muscle cells was extracted six hours after injections [66]. RT-PCR was performed on the muscle extracts, and nuclear receptor-4A1 (Nr4A1) and PPAR coactivator 1 alpha 4 (PGC-1α4) mRNA levels were increased when compared to the control, suggesting a potential role of hordenine in muscle hypertrophic gene expression and positive skeletal muscle function. Hordenine was investigated for its anti-prolactinoma effects in rats, compared to the first-line therapy compound bromocriptine by Wang et al. (2020). The rats were injected in their abdominal cavity with 17α-estradiol (2 mg) to induce prolactinoma, once a day, every five days, for a total of 50 days [95]. Aqueous hordenine was then daily administered intragastrically for 30 days. Hordenine-treated rats saw a reduction in pituitary gland volume that was comparable to bromocriptine when administered with 38.2 mg/kg of hordenine. Rats treated with 76.4 mg/kg and 152.8 mg/kg of hordenine saw an even greater reduction in the volume of their pituitary gland than with bromocriptine. All tested concentrations of hordenine reduced prolactin serum levels, but less than bromocriptine. They concluded that hordenine seems to lessen the effects of prolactinoma through MAPK regulation, by lowering p-p38, p-ERK1/2, and p-JNK protein expression, and diminishing TNF-α, IL-6, and IL-1β production [95]. Similarly, hordenine’s anti-inflammatory capacity was investigated in a lipopolysaccharide (LPS)-induced acute lung injury (ALI) model in mice. The mouse model for ALI was established by the intranasal instillation of 1.25 mg/kg of LPS in a phosphate-buffered saline solution. Mice were pretreated with hordenine (10 mg/kg or 15 mg/kg) through intraperitoneal injections for 1 h [96]. Zhang et al. observed a diminution in pro-inflammatory cytokine expression, such as IL-6, IL-1β, and TNF-α. Following the same trend, Su et al. (2022) published an article regarding hordenine’s anti-inflammatory mechanism in a Parkinson’s disease rat model. Neuroinflammation was induced by an injection of 6-hydroxydopamine (3 μL of 4 μg/μL) in the rats’ right substantia nigra compact [97]. Rats were pretreated with hordenine (50 mg/kg) dissolved in distilled water, once a day for three days, by gavage, and for 5 weeks following the neuroinflammation-inducing injection. They found, through immunohistochemistry and Western blotting, that hordenine inhibits the loss of dopaminergic neurons. Furthermore, they brought evidence that hordenine’s anti-neuroinflammatory activity is being mediated, at least in part, by the suppression of microglia activation, which is a considerable contributor to Parkinson’s disease development, through TNF-α, IL-6, COX-2, and iNOS production, and its consequent neuroinflammation [97]. Hordenine was studied in the context of Alzheimer’s disease in rats by Agrawal et al. (2024). AlCl_3_ (175 mg/kg) was used to induce cognitive dysfunctions similar to the ones exhibited in Alzheimer’s disease by oral supplementation for 28 days. Rats were treated with hordenine (25 and 50 mg/kg) or with donepezil (5 mg/kg), a prescription drug used to ameliorate cognitive function [98]. They reported a positive effect of hordenine on cognitive dysfunction comparable with donepezil, especially at higher doses (i.e., 50 mg/kg of hordenine), suggesting a potential therapeutical role of hordenine in Alzheimer’s disease. They also presented evidence that hordenine decreases Il-β1, TNF-α, as well as NF-κB levels in rat brain tissue.

##### Cannabis HCAA In Vivo Activity

HCAA anti-inflammatory activity isolated from a *Celtis Africana* extract was demonstrated in a carrageenan-induced paw edema in rats by Al-Taweel et al. (2012). Wistar rats received oral adminisrtations of either N-trans-coumaroyltyramine, N-trans-caffeoyltyramine, or N-trans-feruloyltyramine (10 mg/kg). They reported, respectively, a 48%, 25%, and 33% inhibition of edema volume when compared to the control and the positive control diclofenac, with a 58% inhibition [99]. Lee et al. (2021) investigated the effects of N-trans-caffeoyltyramine on C57BL/6 J DIO male mice, injected intraperitoneally (200 mg/kg, bidaily for 14 days). They found a diminution in liver weight, concordant with the release of hepatic lipid content, as well as a color shift from yellow (control) to red (caffeoyltyramine-treated), and additional weight measured in epididymal fat pads compared to control, further confirming a lesser fat content in treated livers [81]. These results show the promise of N-trans-caffeoyltyramine in treating NAFLD.

### 4.3. Biological Activity of Cannabis Flavonoids

Cannflavins A and B possess an anti-inflammatory activity 30 times more potent than that of acetylsalicylic acid, by inhibiting the production of prostaglandins E2 and leukotrienes via microsomal prostaglandin E_2_ synthase-1 (mPGES-1) and 5-lipooxygenase inhibition [15]. Furthermore, cannflavin A exhibits desirable qualities, in that it only weakly inhibits cyclooxygenases COX-1 and COX-2, thereby reducing the negative side effects of COX-inhibiting anti-inflammatory drugs, such as gastritis [100]. Cannflavin A also has potential in treating neurodegenerative disorders. Cannflavins are also useful, in varying degrees, for antiparasitic treatments. The antiparasitic effects have been studied both in vitro and in silico [100]. Cannflavins exhibit anti-nociceptive activity in nematodes and demonstrate antiproliferative activity. Cannflavin A holds promise for anti-viral treatments as well, as in silico studies demonstrate.

#### 4.3.1. Cannabis Flavonoids In Vitro Activity

Cannflavin A was tested by Barrett et al. (1985) against synovial cells for prostaglandin E2 (PE2) inhibition activity. They used phorbol 12-myristate 13-acetate (PMA) (10 ng/mL) as a PE2-inducing compound and reported an IC_50_ = 31 ng/mL, compared to aspirin (IC_50_ = 840 ng/mL) [101]. Cannflavin A was reported to inhibit the TLR4-induced production of IL-1β (IC_50_ = 12.9 μM) and CXCL10 (IC_50_ = 43.4 μM), further corroborating its anti-inflammatory potential [102]. The anti-leishmanial activity of cannflavin A was reported, with an IC_50_ = 4.5 μg/mL [103]. Cannflavin B and C were reported to be anti-leishamanial as well, with an IC_50_ = 5.0 μg/mL, and cannflavin C with an IC_50_ = 17.0 μg/mL [104]. Salem et al. (2011) also tested cannflavin A extracted from *Mimulus bigelovii* for antileishmanial activity, and for antitrypanosomal activity. They report an IC_50_ = 14.6 μg/mL against *Leishmania donovani*, and an IC_50_ = 1.9 μg/mL against *Trypanosoma brucei brucei* [104]. Cannflavin A was tested against PC12 cells by Eggers et al. (2019) for the inhibition of amyloid β fibrillization and neurotoxicity. They reported a protective effect of PC12 cells at 10 μM of cannflavin A, but at higher concentrations, the compound exhibited neurotoxic behavior [105]. At 100 μM of cannflavin A, there was a significant reduction in amyloid β fibrils, suggesting a potential utilization in Alzheimer’s disease prevention. Cannflavin A showed antiproliferative activity against two bladder cancer cell lines (T24 and TCCSUP) at 100 μM after 48 h treatment [106]. Treatment with cannflavin A at 2.5 μM for 24 h, whilst not affecting bladder cancer cell viability, was able to induce caspase 3 cleavage, suggesting it could affect bladder cancer cell apoptosis. Tomko and colleagues also provided evidence that 2.5 μM of cannflavin A could reduce T24 cell invasion in a Matrigel invasion assay, from 25.3% in the control, to 15.1%. Kynurenine 3-monooxygenase (KMO) is a drug target for neuroinflammatory and neurodegenerative diseases. Cannflavin A, extracted from hemp aerial parts, was proven as a KMO inhibitor, with an IC_50_ = 29.4 μM, compared to the positive control Ro 61-8048 (IC_50_ = 5.1 μM), suggesting a possible therapeutical usage in neurodegenerative diseases [107]. Cannflavin A and B diminish lipid peroxidation at concentrations of 1.25 μM and increase cell viability of HaCaT cells treated with erastin, a ferroptosis-inducing molecule, at 5 μM for cannflavin A and 2.5 μM for cannflavin B [108]. This cytoprotective effect is thought to be linked to cannflavins’ antioxidant capabilities. Cannflavin A and B showed anti-nociceptive activity in *Caenorhabditis elegans* in a heat-avoidance assay at concentrations of 1 μM (1 h treatment) [109]. Furthermore, Lahaise et al. (2024) provided evidence that this anti-nociceptive effect dissipated after 6 h, suggesting a rapid onset and offset of cannflavins’ effect.

#### 4.3.2. In Silico Predicted Activity of Cannabis Flavonoids

Cannflavin A has not been investigated in vitro for anti-viral activities, but preliminary in silico studies indicate a relatively high affinity for HIV-1 protease (−9.7 kcal/mol), which is required for infection [110]. For the dengue virus, it presents a high affinity for its envelope protein (−125.7 kJ/mol) [111]. Finally, Zika virus was also tested, and cannflavin A is predicted to have a high affinity and efficient positioning for the virus’ helicase (RNA binding site) (−131.7 kJ/mol), for the ATP binding site of the helicase (−139.8 kJ/mol), the methyltransferase (−126.9 kJ/mol), and the RNA-dependent RNA polymerase (−120.3 kJ/mol) [100,112].

### 4.4. Biological Activity of Cannabis Stilbenes

Stilbenes are thought to be involved in both the constitutive and induced defense of plants; as they possess antimicrobial activities, they also act as a deterrent against fungi, herbivores, and insects, while serving as allelochemicals [12]. *Cannabis* stilbenes present antiproliferative, anti-inflammatory, and antioxidant activity, as well as possible employment for the treatment of diabetes and fat-related diseases. All in all, it seems that stilbenes in *Cannabis* are good antioxidants, anti-inflammatories, and anti-cancer agents, with some additional peculiar effects that would require more attention.

#### 4.4.1. In Vitro Activity of Cannabis Stilbenes

**Figure 16:**
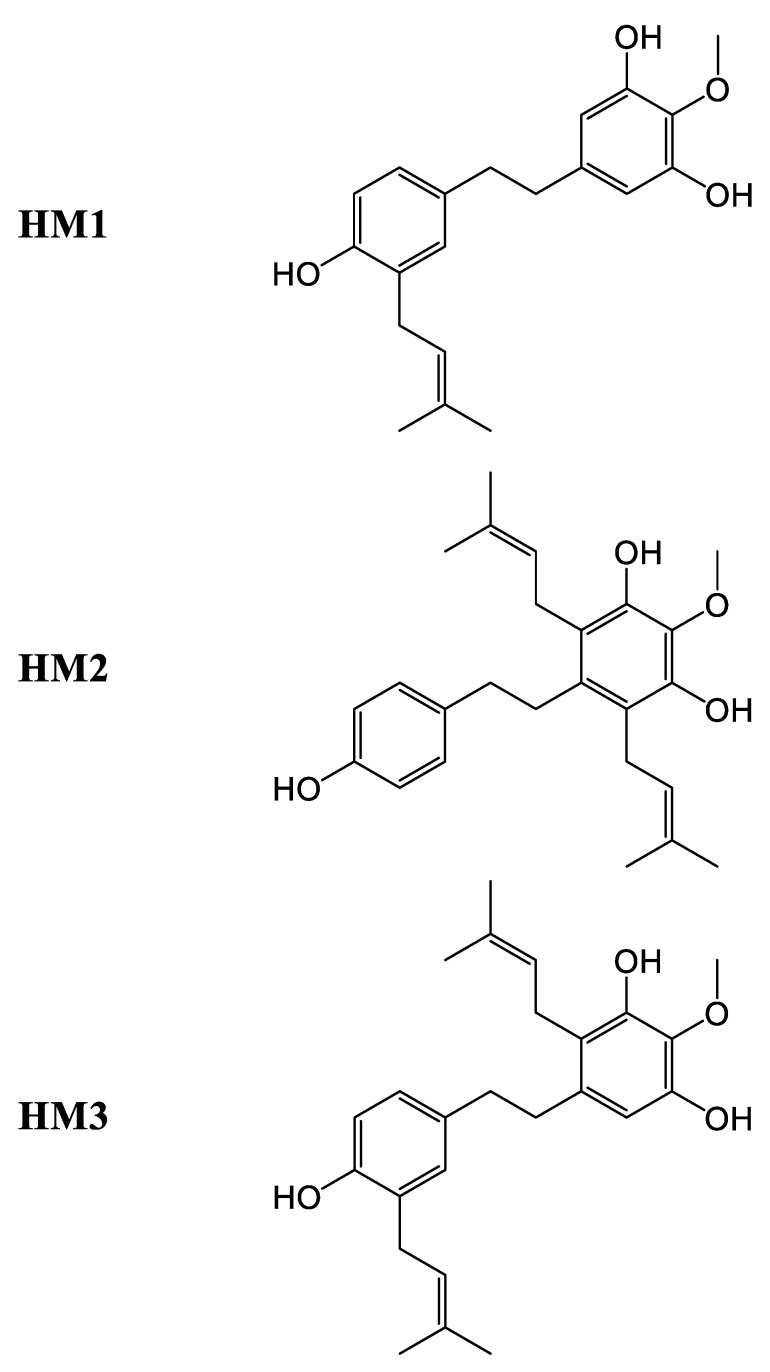
Chemical structure of *Cannabis* stilbenes HM1, HM2, and HM3.

Denbinobin possesses antiproliferative activity against A549 cells (lung adenocarcinoma) treated with 10 and 20 μM for 24 h (48.4% and 56.1% decrease in cell viability), due to apoptosis by Akt inactivation and Bad phosphorylation [113]. Denbinobin also exhibited significant antiproliferative activity against HT-29 and HCT-116 (colon cancer) cell lines at 10 μM [114]. Denbinobin demonstrated pro-oxidant and pro-apoptotic activity against human leukemic cell lines by inactivating the NF-κB pathway, and anti-HIV activity by inhibiting PMA- (IC_50_ = 1.5 μM), TCR/CD28 binding- (IC_50_ = 2.5 μM), and TNF-α-induced (IC_50_ =< 1 μM) LTR transcriptional activity [115]. Denbinobin’s antiproliferative activity was tested against SK-Hep-1 (hepato carcinoma), SNU-484 (gastric cancer), and HeLa (cervical cancer), and it possesses an IC_50_ of 16.4, 7.9, and 22.3 µM, respectively [116]. Moreover, 1–20 μM of denbinobin significantly reduced the invasiveness of SNU-484 cells in a dose-dependent manner (more than 40% reduction at 1 μM). HSC-T6 (rat liver stellate cells) treated with denbinobin (10, 30, and 50 μM) showed significant reduction in cell proliferation in a dose- and time-dependent manner [117]. Denbinobin is effective against the proliferation of two human glioblastoma cell lines (GBM8401 and U87MG), in a dose-dependent fashion at 1–3 μM, via the downregulation of the IKKα–Akt–FKHR signaling pathway [118]. Lu et al. (2014) tested whether denbinobin can be helpful in preventing prostate cancer migration using PC3 cells. They recorded an IC_50_ = 7.5 μM for 24 h denbinobin treatment, with a diminution of 80% in cell viability at 10 μM [119]. Additionally, denbinobin reduced PC3 cell migration at the tested concentrations (5 and 10 μM) by inhibiting CXCL12, which in turn activates Rac1, contributing to lamellipodia formation, which is necessary in metastasis, suggesting that denbinobin could be useful in treating prostate cancer and preventing metastasis. Denbinobin also presents potent anti-inflammatory activity, where it reduces iNOS and COX-2 expression and levels in RAW264.7 cells at 3 μM [120]. Conjointly, denbinobin suppressed NF-κB activation and lowered TNF-α, IL-10, and IL-1β mRNA expression. Canniprene, a dihydrostilbene unique to *Cannabis* as of yet, has anti-inflammatory activity that results in a potent and dose-dependent inhibition of lipooxygenase 5, even more so than cannflavin A, the most potent of the cannflavins for anti-inflammatory activity, with an IC_50_ of 0.4 μM [57,60]. Cannabispirenone had a much lower IC_50_ of 9.3 μM against lipoxygenase 5 [57]. Canniprene, like cannflavin A, has a dose-dependent inhibitory activity for microsomal prostaglandin E2 synthesis, but less than cannflavin A this time, with an IC_50_ of 10 μM [57]. Canniprene also exhibits antiproliferative activity on four of the human cell lines tested, namely MCF-7 (breast cancer), A549 (lung cancer), HepG2 (liver carcinoma), and HT-29 (colorectal adenocarcinoma), with nearly 90% or more dead cells for the four cell lines, with the weakest effect being observed against A549 at concentrations between 1 and 5 mg/L [18]. Three stilbenes first identified in 2018 by Liu et al., namely α,α′-dihydro-3′,4,5′-trihydroxy-4′-methoxy-3-isopentenylstilbene (HM1), α,α′-dihydro-3,4′,5-trihydroxy-4-methoxy-2,6-diisopentenylstilbene (HM2), and α,α′-dihydro-3′,4,5′-trihydroxy-4′-methoxy-2′,3-diisopentenylstilbene (HM3) (Figure 16), demonstrate slight cytotoxic activities against MCF-7 (breast cancer) and A549 (lung cancer) cell lines, whilst HM1 exhibits strong cytotoxic activity against those cell lines. Moreover, the three stilbenes HM1-3 increased the expression of ABCG1 and SR-B1, which are cholesterol transport proteins, when RAW264.7 cells are treated with 1 mg/L of the compounds, suggesting that these could increase cholesterol efflux from macrophages to high-density lipoproteins (HDL) [19]. Dihydroresveratrol exhibited anti-inflammatory activity, reducing the expression of IL-6, IL-18, and IL-1β mRNA in RAW 264.7 cells, when treated with 50 μM of the compound [18,121]. However, dihydroresveratrol treatment increased the expression of TNF-α, a pro-inflammatory cytokine, while still maintaining an overall anti-inflammatory effect. Dihydroresveratrol nullified the H_2_O_2_-induced (100 μM) downregulation of Nrf2 when tested against HepG2 (liver cancer) cells at 20–40 μM for 48 h [122]. Moreover, a dose-dependent upregulation of phosphorylated AMPKα in response to dihydroresveratrol was reported, suggesting antioxidative properties related to AMPK/SIRT1 signaling. Lam et al. (2023) also provided evidence that dihydroresveratrol could enhance the insulin sensitivity of high-glucose high-insulin-exposed HepG2 and C2C12 cells at a concentration 40 μM by effectively phosphorylating AKT. Dihydroresveratrol deserves to be further studied for its possible anti-diabetic effect, where it has been shown to decrease FABP4 (fatty acid binding protein 4) concentrations in peripheral blood mononuclear cells. FABP4 is thought to contribute to insulin resistance, a feature of type II diabetes, with an association between the latter and high FABP4 concentrations [18]. Dihydroresveratrol was able to diminish FATP2 protein expression when AML12 cells were treated with 1 μM for 18 h, suggesting it could reduce de novo lipid biosynthesis and potentially aid in preventing steatosis [123]. Finally, canniprene was investigated in silico for anti-viral activity against SARS-CoV-2, where it could potentially inhibit viral replication by targeting the Mpro protein, necessary for the production of non-structural viral proteins [18].

#### 4.4.2. In Vivo Activity of Cannabis Stilbenes

Denbinobin (10 mg/kg) injected intraperitoneally exhibited anti-metastatic and antiproliferative activities in BALB/c female mice injected with 4T1-Luc cells [124]. A total of 10 mg/kg of denbinobin treatment also inhibited the phosphorylation of Src, FAK, and paxillin. These results suggest that denbinobin can suppress metastasis and cancer progression by downregulating Src-downstream elements. Denbinobin (25 mg/kg) injected intraperitoneally in a nude mice xenograft model against A549 human NSCLC growth indicated that denbinobin significantly inhibited tumor sizes [125]. To verify their in vitro findings, Tsai and colleagues employed an in vivo mouse Matrigel plug assay and injected the Matrigel, containing IGF-1 with denbinobin (1 and 10 μM), in C57BL/6 mice for 7 days. They reported visible color changes in the control plug, indicating angiogenesis, whilst denbinobin 10 μM showed little to no coloration, and only slight coloration in the denbinobin 1 μM plug [125]. These results suggest that denbinobin could be a useful treatment in metastasis prevention and angiogenesis-related diseases, due to its anti-angiogenic activity. Dihydroresveratrol’s anti-obesity effect was verified in C57BL/6J mice fed with a high-fat diet for 6 weeks. Mice received oral administrations of 40 or 80 mg/kg of dihydroresveratrol for five consecutive days per week for three weeks [122]. Both doses significantly reduced mice body weight and blood glucose levels. RT-qPCR experiments revealed that a high dosage of dihydroresveratrol significantly downregulated Mcp1 expression and upregulated Gck expression. They also confirmed that a high dosage was able to reduce lipid accumulation in the liver and that adipocyte enlargement was observed in the control, further confirming the in vitro observations [122]. Cerulein-induced acute pancreatitis (50 μg/kg/h, for 6 h + 1 LPS injection 7.5 mg/kg, intraperitoneally) caused histological damage in rats, and it was reversed by 10 mg/kg of dihydroresveratrol [126]. A total of 10 mg/kg of dihydroresveratrol was also responsible for an almost 50% reduction in plasma α-amylase levels, as well as reducing myeloperoxidase activity by 35%. Contrary to in vitro experiment reports, 10 mg/kg of dihydroresveratrol significantly reduced TNF-α levels, but corroborates the decrease in IL-1β and IL-6 levels from in vitro experiments. Finally, dihydroresveratrol inhibited NF-κB nuclear translocation in a dose-dependent manner [126].

## 5. Regulatory Considerations for Non-Cannabinoid Compounds

Globally, the regulation of herbal medicinal products, including those derived from *Cannabis*, varies significantly across jurisdictions. Many countries have established guidelines to ensure the quality, safety, and efficacy of plant-based medicines. For instance, regulatory agencies such as the European Medicines Agency (EMA) [127], the U.S. Food and Drug Administration (FDA) [128], and Health Canada [129] provide standards for Good Agricultural and Collection Practices (GACP) and Good Manufacturing Practices (GMP) to ensure consistency in medicinal plant production like *Cannabis*. These guidelines typically cover aspects such as cultivation, harvest, and primary processing. However, specific regulations or permissible limits for individual compounds, such as alkaloids and phenolics in *Cannabis*-derived products, are often not well-defined.

This lack of regulation contrasts with other plant-based products, where certain bioactive compounds are subject to stricter controls. For example, regulatory agencies have established maximum allowable limits for pyrrolizidine alkaloids in herbal medicinal products due to their hepatotoxicity. In some countries, safety assessments have been conducted for specific phenolic compounds, particularly in dietary supplements and food products, to mitigate potential health risks. However, comparable evaluations for alkaloids and phenolics in *Cannabis*-derived products are still limited.

The absence of comprehensive regulatory frameworks for these non-cannabinoid compounds highlights the need for further research into their pharmacological and toxicological properties. Establishing standardized guidelines and permissible limits for these bioactive molecules will be essential for ensuring consumer safety and promoting evidence-based applications of *Cannabis*-derived products. While major cannabinoids such as THC and CBD are subject to regulatory oversight in many countries, the legal status of lesser-known cannabinoids (e.g., cannabigerol, cannabinol, cannabichromene, and tetrahydrocannabivarin) remains largely undefined. In most jurisdictions, there are currently no specific regulatory limits or safety evaluations for these compounds. As the global interest in *Cannabis* expands, regulatory agencies may increasingly focus on developing specific policies that address the full spectrum of bioactive compounds present in the plant.

## 6. Future Research Perspectives

Specialized plant metabolites are highly valued across numerous fields, especially in medicine, where they are used to alleviate or even cure ailments. While this review excludes certain well-known groups of *Cannabis* specialized metabolites such as terpenoids and cannabinoids, it underscores the therapeutic potential of other specialized metabolites in *Cannabis* [130], specifically phenolic compounds (like flavonoids and stilbenes) and nitrogen-containing compounds, including hydroxycinnamic acid amides and their dimers, lignanamides, and alkaloids.

Decades of research centered primarily on cannabinoids—coupled with the historical and, in many regions, ongoing illegal status of *Cannabis*—have hindered the study of these other fascinating metabolites by emphasizing the psychoactive aspect of *Cannabis*. However, the traditional usage of *Cannabis* indicates that only a small proportion of the plant’s utilization concerns its psychoactive properties [11]. Among these underexplored compounds, spermidine-type alkaloids in *Cannabis* are notably overlooked; neither their biosynthetic pathways nor their biological activity have been thoroughly characterized, even after 50 years following their chemical identification. Similarly, lignanamides have attracted little research interest, with scarce and unclear explanations for their biosynthesis, despite some findings demonstrating promising biological activity in vitro. This oversight persists despite their potential significance, both in planta and as therapeutic agents. Lignanamides are diverse in number and structure and share many similar biological activities, including antioxidant, antiproliferative, neuroprotective, and anti-inflammatory activities. *Cannabis* lignanamides are often studied from the seeds, where many of them are found, and this gives credit to hemp seed nutraceutical prowess, potentially helpful for preventing diseases like Alzheimer’s, Parkinson’s, and general neuroinflammation [131]. Furthermore, their antiproliferative capacities in vitro suggest potential in treating numerous cancers, a disease with increasing prevalence. However, reports also mention their presence in *Cannabis* roots, warranting their use in research, especially considering that *Cannabis* alkaloids are also present in the roots. In contrast, *Cannabis* HCAAs are far better studied and understood, in part because of their distribution in many plant species used in traditional medicine. They display many nutraceutical activities, like antioxidant, senomorphic, and anti-inflammatory activities, as well as therapeutical activities like antiproliferative, anti-tyrosinase, antimicrobial, antifungal, and lipophagic activities. Their presumed deterrent activity against insects and herbivores could suggest that analogues of these compounds could be used as alternative pesticides. Phenolic compounds, such as flavonoids and stilbenes, are somewhat better represented in the literature, with well-documented biosynthetic pathways and considerable research into their therapeutic properties in vitro, but not so much in vivo. *Cannabis* flavonoids seem to be an interesting class of molecules, exhibiting impressive anti-inflammatory activity, solid potential in inhibiting and attenuating neurodegenerative diseases and neuroinflammation, antioxidant, antiproliferative, and anti-nociceptive activities. *Cannabis* stilbenes are numerous, but few have been investigated for biological activity, but those that have show similar activity. Among them, the well-known denbinobin has anti-inflammatory, antiproliferative, and anti-metastasis activities. Canniprene also exhibits anti-inflammatory and antiproliferative activities, and cannabispirenone exhibits anti-inflammatory activity. The similar activity of *Cannabis* stilbenes is peculiar, which may be indicative of a lack of exploration in their possible activity, or rather, of a mechanism inherent to their structure, possibly indicating that they would be interesting candidates in cancer drug discovery.

The future in *Cannabis* research is sure to require tremendous amounts of work, but also deliver promise [132]. Many things remain unknown or unsure, and future researchers should consider the following to try answering these gaps. Lignanamides have demonstrated worthy activities, but their full potential is surely not met yet. As for alkaloids, the bio-guided fractionation of various tissue extracts would be a decent approach to identify bioactive properties and compounds of such classes. This would apply to stilbenes and flavonoids as well, whose full activity spectrum remains unknown. *Cannabis* stilbenes could be explored for their known antiproliferative activity, as to identify molecular targets, following which molecular docking studies could explore crucial structural factors rendering them bioactive, especially as stilbenes are numerous and processing them one by one would be tedious. This could open the way for stilbenes in cancer drug therapy. Cannflavins have been the subject of in silico studies for anti-viral activity. It would be worthwhile to investigate this presupposed activity in vitro, as to confirm its validity. Furthermore, cannflavins remarkable anti-inflammatory activity could be beneficial to human usage and in vivo studies are what would be required in this step of the process to validate their efficiency and safety. Current challenges in the study of *Cannabis* alkaloids include the lack of information regarding the biosynthetic process, genes involved in biosynthesis, bioactivity, the lack of reasonably priced chemical standards, as well as small concentration levels in planta, the last point being applicable to many NC metabolites, as demonstrated in Table 3. In this same vein, the lack of knowledge regarding metabolomic variations between cultivars and chemotypes (other than cannabinoids) renders research optimization difficult, as in we do not know which cultivar produces the highest titers of a given specialized metabolite to be studied. This could cause researchers additional challenges that could be barred with adequate knowledge acquisition, notably by measuring specialized metabolite levels in various cultivars and chemotypes. It would then be imperative to identify correlations between genetic features and specialized metabolite production, as well as gene identification and characterization, to be able to analyze environmental effects on the transcriptome and metabolome. Furthermore, gene identification and enzyme characterization could permit the study of the specialized metabolites’ role for *C. sativa*, which would deepen our understanding of this plant. Many fronts are to be tackled in the *-omics* sciences regarding *Cannabis*. Bio-guided fractioning for metabolomics could allow metabolite discovery and their pharmacological characterization; transcriptomic studies would allow gene identifications and possibly the linkage of metabolomic profiles to certain cultivars or chemotypes; proteomic studies would allow for a clearer understanding of environmental effects on specialized metabolite production and could even allow for the production of metabolites in heterologous hosts for pharmacological and toxicological studies. It is also worth considering the lack of reference plant models in *Cannabis* research. Exemplified by *Arabidopsis thaliana* in plants, there is no standard cultivar or genetic line for *Cannabis*, making it so that researchers work with what they can obtain, instead of concentrating their efforts into one model to breach the ongoing challenges limiting the speed at which we uncover knowledge about *Cannabis*. Legislative regulations are also of considerable hinderance to researchers, considering that the less concentrated the specialized metabolites are, the more biomass is needed, and in vivo studies with isolated metabolites are needed to further confirm their in vitro activities. The lack of harmony in legislation is also affecting the way researchers can proceed and work together to face said challenges.

## 7. Conclusions

This review highlights the therapeutic potential of non-cannabinoid specialized metabolites in *Cannabis*, focusing on phenolic compounds and nitrogen-containing metabolites such as alkaloids and lignanamides. Despite their diverse pharmacological activities, research on these compounds remains limited, largely due to historical research bias towards cannabinoids, legal constraints, and the absence of comprehensive regulatory frameworks. Future research should prioritize biosynthetic pathway elucidation, bioactivity screening, and metabolomic studies to advance our understanding of these metabolites. Additionally, developing standardized regulatory guidelines for non-cannabinoid compounds in *Cannabis*-derived products will be essential to ensure product safety and facilitate their integration into modern medicine.

## Figures and Tables

**Table 1:** Classes of phenolic compounds and molecular examples found in *C. sativa*.

Type	Example	Structure
Simple phenols	Eugenol	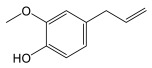
Phenolic acids	Protocatechuic acid	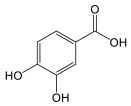
Cinnamic acids	*p*-coumaric acid	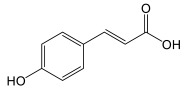
Flavones	Cannflavin B	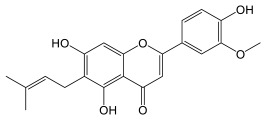
Flavanones	Naringenin	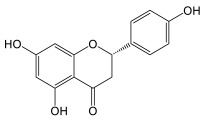
Anthocyanins	Cyanidine 3-*O*-rutinoside	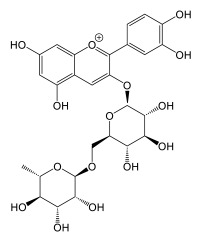
Stilbenes	Dihydroresveratrol	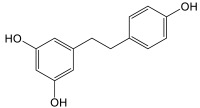

**Table 2:** Non-exhaustive classification of *Cannabis* alkaloids according to their chemical structure and biosynthetic origin.

Type	Precursor	Chemical	Example	Structure
Piperidine	Aspartate/ ornithine	Heterocyclic	Piperidine	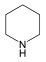
Pyrrolidine	Arginine/ ornithine	Heterocyclic	Pyrrolidine	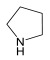
Spermidine	Arginine/ ornithine	Heterocyclic	Cannabisativine	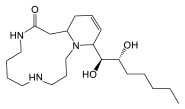
Pyridine	Tryptophan	Heterocyclic	Trigonelline	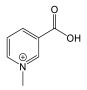
Phenylethyl-amine	Tyrosine/ Phenylalanine	Aliphatic	Hordenine	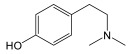

**Table 3:** Portrait of *Cannabis* specialized metabolites levels and their pharmacological significance.

Compound	Range Levels (mg/kg)	Mean Levels (mg/kg)	Cultivar for Quantification	Tissue	Pharmacological Significance	Ref.
Cannabisativine	-	2.5		Root, leaf	-	
Anhydrocannabisativine	-	0.3		Root, leaf	-	[21]
Hordenine	-	-		Root	Antibacterial, anti-asthmatic, smooth muscle relaxation, anti-inflammatory, muscle hypertrophy stimulation, anti-prolactinoma, neuroprotection, cognitive dysfunction attenuation	
*N*-*p*-*trans*-coumaroyltyramine	7.6–19.8 -	11.8 196.9	High THC variety High CBD variety Intermediate variety Zenit	Root, seed, leaf	Antiproliferative, tyrosine kinase inhibition, antiproliferative drug sensitivity enhancer, acetylcholinesterase inhibition, antioxidant, senomorphic activity, antibacterial, insect deterrent, anti-melanogenesis	[82,131]
*N*-caffeoyltyramine	0.1–76.2 -	23.7 339.0	Antal Carmagnola Kompolti Tiborszallasi Zenit	Inflorescence, seed, root	Antiproliferative, tyrosine kinase inhibition, neuroprotective, antioxidant, anti-inflammatory, lipophagic agent, antifungal, anti-melanogenesis	[51,131]
*N*-feruloyltyramine	-	278.8	Zenit	Seed, root, leaf	Antiproliferative, tyrosine kinase inhibition, neuroprotective, antioxidant, anti-inflammatory, senomorphic activity, lipophagic agent, insect deterrent, anti-melanogenesis	[131]
Grossamide	-	830.0	Zenit	Seed, root	Anti-inflammatory, neuroprotective	[131]
Cannabisin A	0.005–2.9 -	1.0 82.7	Antal Carmagnola Kompolti Tiborszallasi Zenit	Inflorescence, seed	Antioxidant, tyrosinase inhibition	[51,131]
Cannabisin B	0.02–1.1 -	0.5 130.0	Antal Carmagnola Kompolti Tiborszallasi Zenit	Inflorescence, seed, root	Antioxidant, antiproliferative, tyrosinase inhibition	[51,131]
Cannabisin C	0.003–0.4 -	0.1 179.7	Antal Carmagnola Kompolti Tiborszallasi Zenit	Inflorescence, seed, root	-	[51,131]
Cannabisin D	-	444.4	Zenit	Seed	Antioxidant, anti-inflammatory	[131]
Cannabisin F	-	471.0	Zenit	Seed	Anti-inflammatory, neuroprotective	[131]
Cannabisin G	-	-		Root	Antioxidant	
Cannabisin Q	-	201.3	Zenit	Seed	-	[131]
Cannflavin A	19.6–130.0 21–280	61.8 97	Antal Carmagnola Kompolti Tiborszallasi Ermo Carma THC-3 MH-WU-112	Inflorescence, leaf	Anti-inflammatory, anti-leishmanial, anti β-amyloid, antiproliferative, KMO inhibition, lipid peroxidation inhibition, anti-nociceptive, potential anti-viral activity	[51,57]
Cannflavin B	11.9–215.5 9–106	84.5 43	Antal Carmagnola Kompolti Tiborszallasi Ermo Carma THC-3 MH-WU-112	Inflorescence, leaf	Lipid peroxidation inhibition, anti-nociceptive	[51,57]
Cannflavin C	-	-		Leaf	Anti-leishmanial	
Denbinobin	-	-		Leaf	Antiproliferative, anti-metastatic, anti-inflammatory	
Canniprene	4–2085	433	Ermo Carma Carmagnola THC-3 MH-WU-112	Leaf	Anti-inflammatory, antiproliferative, potential anti-viral activity	[57]
Cannabispirenone	-	-		Leaf	Anti-inflammatory	
HM1	-	-		Leaf	Antiproliferative, cholesterol transport	
HM2	-	-		Leaf	Antiproliferative, cholesterol transport	
HM3	-	-		Leaf	Antiproliferative, cholesterol transport	

For level comparison, cannabinoids such as THCA can attain concentrations of 300,000 mg/kg (30% ^m^/_m_) in flowers. According to the SeedFinder database, Antal is an indica-dominant low-THC (0,2%) moderate-CBD (8%) hemp cultivar. Carmagnola is a sativa-dominant low-THC (0.5%) moderate-CBD (4%) hemp cultivar. Kompolti is a sativa-dominant no-THC, high-CBD (14%) hemp cultivar. Tiborszallasi is a low-THC (<0.2%) low-CBD (2–3%) hemp cultivar. Zenit is a sativa-dominant low-THC (<0.02%) low-CBD (0.5%) hemp cultivar. According to [57], cultivars Ermo, Carmagnola, and MH-WU-112 are hemp fiber and CBD-chemotypes cultivars. Carma is hemp fiber CBG-chemotype cultivar. THC-3 is a narcotic THC-chemotype cultivar. Their details were not found on the SeedFinder database.

## Data Availability

No data generated, not applicable.

## Appendix A

This appendix section contains tables that provide a comprehensive list of *Cannabis* genes identified in the NCBI that are involved in key metabolic pathways. Table A1 presents genes associated with the biosynthesis of the aromatic amino acids phenylalanine and tyrosine, which serve as precursors for numerous specialized metabolites. Table A2 details genes involved in the phenylpropanoid pathway, including those responsible for the production of cinnamic acids and their amides, essential intermediates in lignin and flavonoid biosynthesis. Table A3 lists genes encoding enzymes for the biosynthesis of polyamines (spermine and spermidine) and spermidine-derived alkaloids, which play vital roles in plant growth and stress responses. Finally, Table A4 and Table A5 highlight genes involved in flavonoid and stilbene biosynthesis pathways, respectively, which are pathways crucial for the production of bioactive compounds with diverse physiological and pharmacological properties.

The sequencing of the *Cannabis* genome, from which the genes mentioned below are sourced, was carried out by Harm van Bakel’s team in 2011 [133]. One ADH1 gene (LOC133033635) is not from the article produced by van Bakel et al. (2011).

**Table A1:** Listed *Cannabis* genes found on NCBI involved in the biosynthesis of the aromatic amino acids phenylalanine and tyrosine.

Enzyme	Name	Gene ID	Chromosome	mRNA	Protein	Isoform
Chorismate mutase	CM1	LOC115700776	8	XM_030628416.2	XP_030484276.1	X1
				XM_030628417.2	XP_030484277.1	X2
	CM2	LOC115699048	8	XM_030626256.2	XP_030482116.2	-
		LOC115694782	X	XM_030621875.2	XP_030477735.2	X1
				XM_030621874.2	XP_030477734.2	X2
Prephenate amino-transferase	PPA-AT	LOC115712503	4	XM_030640790.2	XP_030496650.2	-
		LOC115712504	4	XM_030640791.2	XP_030496651.2	-
Arogenate dehydratase	ADT1	LOC115698788	8	XM_061102953.1	XP_060958936.1	X1
				XM_030625964.2	XP_030481824.2	X2
				XM_061102954.1	XP_060958937.1	X3
				XM_061102955.1	XP_060958938.1	X4
				XM_061102956.1	XP_060958939.1	X5
	ADT2	LOC115721090	2	XM_030650332.2	XP_030506192.1	X1
				XM_030650334.2	XP_030506194.1	X2
				XM_030650336.2	XP_030506196.1	X3
	ADT3	LOC115719371	2	XM_030648387.2	XP_030504247.1	-
Arogenate dehydrogenase	ADH1	LOC115703598	1	XM_030630828.2	XP_030486688.2	-
		LOC133033635	1	XM_061108628.1	XP_060964611.1	-
		LOC115725067	6	XM_030654482.2	XP_030510342.1	-
	ADH2	LOC115707881	1	XM_030635972.2	XP_030491832.2	-

**Table A2:** Listed *Cannabis* genes found on NCBI involved in the phenylpropanoid pathway and in the biosynthesis of cinnamic acids and their amides.

Enzyme	Name	Gene ID	Chromosome	mRNA	Protein
Phenylalanine ammonia-lyase	PAL1	LOC115706668	1	XM_030634382.2	XP_030490242.2
	PAL	LOC115709862	3	XM_030638093.2	XP_030493953.1
		LOC115709383	3	XM_030637471.2	XP_030493331.2
Cinnamate 4-hydroxylase	C4H	LOC115719463	2	XM_030648524.2	XP_030504384.1
	CYP73A100	LOC115709609	3	XM_030637745.2	XP_030493605.2
4-Coumarate-CoA ligase	4CL1	LOC115699364	8	XM_030626724.2	XP_030482584.2
	4CL2	LOC115717276	5	XM_030646244.2	XP_030502104.2
	CCL1	LOC115694777	6	XM_061116895.1	XP_060972878.1
				XM_030621866.2	XP_030477726.2
Caffeoyl-CoA-*O*-methyltransferase	CCOMT	LOC115702947	X	XM_030630419.2	XP_030486279.1
Caffeate 3-*O*-methyltransferase	COMT	LOC115716906	5	XM_030645827.2	XP_030501687.2
		LOC115716367		XM_030645149.2	XP_030501009.2
		LOC115697697	7	XM_030624792.2	XP_030480652.2
		LOC115721930	9	XM_030651036.2	XP_030506896.2
		LOC115723302		XM_030652742.2	XP_030508602.2
Tyrosine decarboxylase	TYDC	LOC115696858	7	XM_030623740.2	XP_030479600.1
		LOC115721923	9	XM_030651029.2	XP_030506889.2
	TYDC1	LOC115721880	9	XM_061105012.1	XP_060960995.1
Spermidine hydroxycinnamoyl transferase	SHCT	LOC115716016	5	XM_030644703.2	XP_030500563.2
		LOC115695248	6	XM_030622328.2	XP_030478188.2
		LOC115696820	7	XM_030623703.2	XP_030479563.2
Spermidine hydroxycinnamoyl Transferase-like	SHCT-L	LOC115712649	4	XM_030640956.2	XP_030496816.2
		LOC115716993	5	XM_030645919.2	XP_030501779.2
		LOC115716541		XM_030645353.2	XP_030501213.2
Hydroxycinnamoyl transferase	HCT	LOC115721023	2	XM_030650250.2	XP_030506110.2

Other sequences corresponding to phenylalanine ammonia lyase-like enzymes are also found in the NCBI, but these genes are not listed in the table above. The Cs4CL4 mentioned in Section 3.6 corresponds to 4CL2 in Table A2. The caffeate 3-*O*-methyltransferase (COMT) genes encode for the enzymes capable of producing ferulic acid, by methylating caffeic acid. They are numerous in *Cannabis,* and they have notable variations between them at the protein level. This suggests that this gene has been the subject of duplication over time and that it is probably expressed in various tissues and with different regulations. We also find many COMT genes in the NCBI, with comparable sequences that have not yet been characterized. The search for spermidine-hydroxycinnamoyl transferases in the NCBI gives several results, including spermidine coumaroyl-CoA acyltransferases and proteins that are spermidine-hydroxycinnamoyl transferase-like. These are listed in Table A2. It is likely that there are many of these spermidine-hydroxycinnamoyl transferase-like proteins, as they have not yet been characterized enzymatically, and do not necessarily use spermidine as a substrate. There is no isoform listed for the genes in Table A2.

**Table A3:** Listed *Cannabis* genes found in the NCBI involved in the biosynthesis of the polyamines spermine and spermidine, spermidine alkaloid.

Enzyme	Name	Gene ID	Chromosome	mRNA	Protéine	Isoform
Ornithine decarboxylase	ODC	LOC115714249	X	XM_030642880.2	XP_030498740.1	-
Arginine decarboxylase	ADC	LOC115725364	6	XM_030654854.2	XP_030510714.2	-
Agmatine Imino-hydrolase	AIH	LOC115710158	3	XM_030638493.2	XP_030494353.2	-
*N*-carbamoyl-putrescine amidase	CPA	LOC115721138	2	XM_030650390.2	XP_030506250.2	-
*S*-adenosyl-methionine synthase	SAMS1	LOC115711364	X	XM_030639731.2	XP_030495591.1	-
		LOC115719259	2	XM_061108853.1	XP_060964836.1	X1
				XM_061108854.1	XP_060964837.1	X2
				XM_030648224.2	XP_030504084.1	X3
	SAMS3	LOC115711343	3	XM_030639680.2	XP_030495540.1	-
SAM decarboxylase proenzyme	SAMDC	LOC115725611	6	XM_030655195.2	XP_030511055.2	-
	SAMDC4	LOC115721913	9	XM_030651018.2	XP_030506878.1	-
Spermidine synthase	SPDS	LOC115716589	4	XM_030641228.2	XP_030497088.2	-
Spermine synthase	SPS	LOC115716589	5	XM_030645419.2	XP_030501279.2	X1.1
				XM_061115311.1	XP_060971294.1	X1.2
				XM_061115312.1	XP_060971295.1	X2.1
				XM_030645418.2	XP_030501278.2	X2.2
*N*-myristoyl-transferase	NMT	LOC115707577	1	XM_030635591.2	XP_030491451.1	-

Very few enzymes involved in the biosynthesis of cannabisativine and anhydrocannabisativine are known or available, with the exception of polyamine biosynthesis genes. Table A3 shows the gene for an enzyme that adds a myristoyl group to a glycine at the N-terminus of a protein, possibly capable of catalyzing the condensation reaction between a spermidine molecule and a myristic acid. Polyamine biosynthesis genes are well-conserved across species and are therefore easily characterized. Table A3 shows all the genes required for spermine and spermidine biosynthesis.

**Table A4:** Listed *Cannabis* genes found in the NCBI involved in flavonoid biosynthesis.

Enzyme	Name	Gene ID	Chromosome	mRNA	Protein	Isoform
Chalcone synthase	CHS	LOC115724170	9	NM_001397940.1	NP_001384869.1	-
Chalcone isomerase	CHI	LOC115716382	5	XM_030645165.2	XP_030501025.2	X1
				XM_061115784.1	XP_060971767.1	X2
				XM_061115783.1	XP_060971766.1	X3
				XM_061115782.1	XP_060971765.1	X4
Flavanone 3-dioxygenase	F3H	LOC115702709	-	XM_030630171.2	XP_030486031.2	-
Flavonol synthase	FLS	LOC115717395	5	XM_030646361.2	XP_030502221.2	-
Dihydroflavonol 4-reductase	DFR	LOC115710150	3	XM_030638485.2	XP_030494345.2	-
Leucoantho-cyanidin dioxygenase	LDOX	LOC115716756	5	XM_030645652.2	XP_030501512.2	-
UDP-glucose–flavonol *O*-glucosyl-transferase	UFGT	LOC115703032	X	XM_061106990.1	XP_060962973.1	-
		LOC115704110	X	XM_030631332.2	XP_030487192.2	-
		LOC115704783	X	XM_061106847.1	XP_060962830.1	-
		LOC115704809	1	XM_030632016.2	XP_030487876.2	-
Flavonoid 3′-hydroxylase	F3′H	LOC115715620	5	XM_030644276.2	XP_030500136.2	-
SAM-dependent-methyltransferase	SAM-MT	LOC115724992	6	XM_030654382.2	XP_030510242.1	X1
				XM_061117310.1	XP_060973293.1	X2

Known genes involved in *Cannabis* flavonoid biosynthesis are presented in Table A4. Almost all enzymes presented in Figure 12 and Figure 13 are present, with the exception of flavone synthase I and II (FNSI/II), isoflavone synthase (ISF), and prenyl- and geranyltransferase enzymes. Note that the chromosomal location of the F3H gene is unknown.

**Table A5:** Listed genes of *Cannabis* found in the NCBI involved in stilbene biosynthesis.

Enzyme	Name	Gene ID	Chromosome	mRNA	Protein	Isoform
2 alcenal reductase	DBR	LOC115708061	1	XM_030636233.2	XP_030492093.2	-
		LOC115697048	7	XM_030623951.2	XP_030479811.1	-
		LOC115697049		XM_030623952.2	XP_030479812.1	-
		LOC115698188		XM_030625376.2	XP_030481236.1	-
		LOC115696848		XM_030623733.2	XP_030479593.1	-
		LOC115697314		XM_030624260.2	XP_030480120.1	X1
				XM_061118868.1	XP_060974851.1	X2
				XM_061118869.1	XP_060974852.1	X3
Bibenzyl synthase	BBS	LOC115713254	X	XM_030641739.2	XP_030497599.2	-

Known genes involved in stilbene biosynthesis in *Cannabis* are identified in Table A5. There are six copies of the double bond reductase (DBR). The bibenzyl synthase (BBS) in Table A5 corresponds to the CsBBS2 presented in the Section Biosynthesis of Cannflavins.
